# Global diversities of disability caused by breast cancer in 204 countries and territories: a secondary analysis from global burden of disease study 2021

**DOI:** 10.1186/s12889-026-27922-z

**Published:** 2026-06-06

**Authors:** Zhen-feng Zhu, Ming-yuan Wu, Wen-chang Jia, Bing-hong Chen, Lei Wu, Xiang-ting Fu, Xi-xi Gu, Xiao-pan Li

**Affiliations:** 1 Departmentof Integrative Medicine, Zhongshan Hospital, Fudan University, Shanghai, 200032 China; 2 Department of Health Management Centre, Zhongshan Hospital, Fudan University, 137 Longquanshan south Rd, Shanghai, 201600 China; 3 School of Public Health, Fudan University, Shanghai, China; 4 The Second Clinical Medical College of Henan University of Chinese Medicine, Zhenzhou, Henan China; 5 Shanghai Key Laboratory of Meteorology and Health, Shanghai, China

**Keywords:** Breast cancer, Global diversities, Trend analysis, Burden of disease

## Abstract

**Background:**

Breast cancer (BC) has become the most common malignancy worldwide. Our aim was to assess the global diversity of disability due to breast cancer in 204 countries and territories.

**Methods:**

Using data from the Global Burden of Disease (GBD) 2021 study the diversity of disability due to breast cancer was estimated for 204 countries and territories, and breast cancer incidence, prevalence, mortality, and Years Lived with Disability (YLDs) were further assessed. Absolute number, rate, and age standardized rate with 95% uncertainty intervals (UIs) were used to compare the difference. Data analysis was further stratified by the socio-demographic index (SDI) of the regions, average annual percentage changes (AAPC) and trends were analyzed by the Joinpoint regression procedure.

**Results:**

From 1990 to 2021, the global disability-adjusted life years (DALYs) caused by breast cancer showed a trend of first decrease and then increase in 204 countries, especially during the epidemic period of Corona Virus Disease 2019 (COVID-19) from 2019 to 2021, with 63% of countries experiencing an increase in DALYs for three consecutive years, mainly in Africa. During this period, there was a significant increase trend in DALYs caused by breast cancer in low - and middle-income countries, with Turkey having the highest growth rate. High-income countries showed declines, with Denmark, Luxembourg and the United Kingdom experiencing declines of more than 50%. For males, the highest incidence, prevalence, mortality, and DALYs are mainly concentrated in the Eastern Sub-Saharan Africa region. For females, high incidence and prevalence are found in economically developed regions such as Europe and North America, while the highest mortality and DALYs are primarily concentrated in the Oceania region. The DALYs of breast cancer patients in the 15–49 years age group are highest in low-middle SDI countries, approximately 180.86 per 100,000 (95% UI:161.08, 201.32). For the 50–74 years age group, the highest DALYs are found in low SDI countries, about 754.47 per 100,000 (95% UI: ). In the ≥ 75 years age group, the highest DALYs are observed in high SDI countries, approximately 1166.46 per 100,000 (95% UI:952.49, 1296.24). Overall, DALYs showed a slight global increase from 2012 to 2021 but a decrease over the 30-year span from 1990 to 2021.

**Conclusions:**

From 1990 to 2021, DALYs due to breast cancer worldwide decreased first and then increased, especially during the COVID-19 pandemic. There is a significant increase in breast cancer DALYs in low - and middle-income countries, while there is a downward trend in high-income countries. In addition, there is a complex relationship between breast cancer indicators and socio-demographic index (SDI) in different countries and regions, indicating the importance of considering multiple factors when analyzing cancer epidemiology and the need to tailor cancer prevention and treatment approaches based on local context and social characteristics.

**Supplementary Information:**

The online version contains supplementary material available at 10.1186/s12889-026-27922-z.

## Introduction

Breast cancer, a leading cause of morbidity and mortality among women worldwide, often leaves survivors facing a range of physical, psychological, and social disabilities [1–3]. Despite advances in treatment and diagnosis, the disabling impact of breast cancer remains a significant public health concern [4–6]. To develop effective strategies to mitigate this impact, a comprehensive understanding of the prevalence, distribution, and predictors of breast cancer related disabilities across diverse socio-economic and cultural contexts is imperative [7, 8].

This secondary analysis, drawing from the Global Burden of Disease Study 2021, aims to fill this knowledge gap by providing an accurate assessment of the global diversities of disability caused by breast cancer in 204 countries and territories [9, 10]. By estimating disability-adjusted life years (DALYs) [11] as a metric, this study captures not only the years of life lost due to premature mortality but also the years lived with disability.

Years Lived with Disability (YLDs) represent the number of healthy years lost due to non-fatal health outcomes from breast cancer. YLDs are computed as prevalence multiplied by the disability weight reflecting disease severity. The Socio-demographic Index (SDI) is a composite indicator of development status strongly correlated with health outcomes. It comprises three components, calculated using geometric mean: total fertility rate under the age of 25, mean education for individuals aged ≥ 15 and lag distributed income per capita. In the GBD 2021, countries and territories were stratified into five SDI categories: low, low middle, middle, high middle and high. Notably, lower SDI values indicate lower levels of societal development [12].

The analysis further identifies key patterns and predictors of breast cancer related disabilities, considering a range of socio-economic, cultural, and health system factors [13–15].

This study advances existing research in four key aspects: [1] It provides 32-year continuous trends (1990–2021) of breast cancer disability burden (YLDs/DALYs) across 204 countries and territories, offering the longest time-span global analysis of breast cancer-related disability to date; [2] It is the first study to systematically quantify the impact of the COVID-19 pandemic on global breast cancer disability burden and its regional heterogeneity; [3] It simultaneously analyzes male and female breast cancer burden, and reveals the significant interaction pattern between age and SDI on breast cancer DALYs, which has been rarely reported in previous studies; [4] It identifies the heterogeneous driving factors of breast cancer burden between high-SDI and low-SDI regions, providing direct evidence for the formulation of precision prevention and control strategies adapted to local development levels.

The findings of this study are crucial for informing evidence-based strategies aimed at improving the quality of life for breast cancer survivors and their families. By evaluating the influence of diverse factors on breast cancer-related disabilities, we hope to gain a deeper understanding of the complex interplay between individual, societal, and environmental factors that contribute to the disabling impact of this disease. Ultimately, this knowledge will contribute towards the development of more targeted and effective interventions, enhancing the lives of those affected by breast cancer globally.

## Methods

### Data sources

The data for this comprehensive secondary analysis on the global disabilities caused by breast cancer were derived primarily from the Global Burden of Disease (GBD) Study 2021 [16]. The GBD Study is an annual effort to quantify health loss due to various diseases, injuries, and risk factors at the global, regional, and national levels [17, 18]. It provides a robust dataset on incidence, prevalence, mortality, and years lived with disability (YLDs) for a wide range of health conditions [19, 20]. For breast cancer specifically, the GBD Study includes detailed information on the number of cases, deaths, and DALYs attributed to the disease. Additionally, we accessed various international databases and repositories such as the World Health Organization (WHO) [21], the International Agency for Research on Cancer (IARC) [22], and the United Nations (UN) to obtain additional contextual information on health systems, healthcare resources, and population-specific factors that may influence the burden of breast cancer and its associated disabilities.

The 21 geographical regions for 204 countries/territories in this study fully adopt the standard geographic classification framework officially released by the GBD 2021 study. This classification is formulated based on the similarity of geographical location, socio-economic development characteristics, and epidemiological features, and is widely used in global disease burden-related research. The specific list of 21 regions is: Central Asia, Central Europe, Eastern Europe, Australasia, High-income Asia Pacific, High-income North America, Southern Latin America, Western Europe, Andean Latin America, Caribbean, Central Latin America, Tropical Latin America, North Africa and Middle East, South Asia, East Asia, Oceania, Southeast Asia, Central Sub-Saharan Africa, Eastern Sub-Saharan Africa, Southern Sub-Saharan Africa, Western Sub-Saharan Africa.

### Estimation

This study provides a comprehensive analysis of incidence, prevalence, mortality, YLDs, and DALYs caused by breast cancer. In terms of data processing, we calculated the age standardization rate of these health indicators based on the global age structure from 2021 to ensure the accuracy and comparability of the results. First, we extracted the GBD Study 2021 data on breast cancer-related YLDs, which measures the loss of healthy life due to the disease. YLDs are calculated by multiplying the prevalence of a condition by its disability weight, which reflects the relative severity of the condition. Next, we used the Bayesian meta-regression model (DisMod-MR 2.1), the core model of the GBD study framework, statistical models to adjust the YLDs for population-specific factors such as age, sex, and socioeconomic status [23]. These models were developed based on previous epidemiological studies and known associations between these factors and breast cancer outcomes, and have been widely validated in GBD studies to address data heterogeneity, missing data, and non-representative sampling across different countries and regions [16]. Finally, we applied spatial smoothing and geospatial mapping techniques based on the R packages ‘ggmap’ and ‘ggplot2’ geospatial analysis techniques to map the estimated disabilities caused by breast cancer across the 204 countries and territories included in the study. This allowed us to identify regional and country-specific patterns and variations in the burden of breast cancer-related disabilities. In this study, we adjusted the age-standardized rates using the direct method and the World standard population accounting method to eliminate the effect of differences in population age structure on the results. We strictly follow the accurate and transparent Guidelines for Accurate and Transparent Health Estimates Reporting (GATHER) to ensure scientific and reliable research [24].

All analyses related to biological differences in this study are based on the sex variable (male/female) from the GBD 2021 study, which is a biological classification consistent with the GBD data collection standard.

### Statistical analyses

On this basis, this study intends to conduct quantitative analysis of core breast cancer indicators including incidence, mortality, prevalence, YLDs, and DALYs, which reflect the whole process from onset to survival and death of breast cancer. This method can not only realize the comparison between different periods, different countries and different regions, but also carry out different statistical analysis of people of different ages, so as to obtain more accurate and comparable evaluation results. The age group was divided into three groups: 15–49 years old, 50–74 years old, and over 75 years old. The three age groups (15–49, 50–74, ≥ 75 years) follow standard GBD study conventions widely used in global burden analyses. This grouping reflects reproductive age patterns, clinical screening periods, and geriatric health characteristics commonly adopted in international cancer epidemiology [25]. On this basis, different regions are classified into five grades based on the SDI: high, high-middle, middle, low-middle, low [22]. The five SDI grades adopt the official 5-stratification standard of GBD 2021, with specific definitions as follows: High SDI: Countries/territories with SDI values in the top 20% of the world, mainly high-income countries in Europe and America, high-income Asia Pacific, etc., with a complete medical system, high per capita income and high education level. High-middle SDI: Countries/territories with SDI values in the 20%-40% quantile of the world, mostly newly industrialized countries, with a rapidly developing economy and medical system. Middle SDI: Countries/territories with SDI values in the 40%-60% quantile of the world, with medium development level and medium level of medical resources and health security system. Low-middle SDI: Countries/territories with SDI values in the 60%-80% quantile of the world, mostly low- and middle-income countries, with limited economic development level and relatively scarce medical resources. Low SDI: Countries/territories with SDI values in the bottom 20% of the world, mainly low-income countries in sub-Saharan Africa, with backward economic development, serious shortage of medical resources, and weak health security system. Average Annual Percent Change (AAPC) was used to show the trends of disease burden from 1990 to 2021 and was calculated by the Joinpoint Regression Program (4.9.0.0, March 2021, Statistical Methodology and Applications Branch, Surveillance Research Program, National Cancer Institute, Bethesda, MD, USA). Joinpoint regression was performed based on the age-standardized rates of breast cancer DALYs per 100,000 population, which were standardized using the GBD global standard population structure, to eliminate the impact of population age structure differences on trend analysis. The maximum number of joinpoints was set to 3, which is the default setting of the Joinpoint Regression Program and widely used in GBD-related long-term trend analysis, to avoid overfitting of the model while capturing the significant inflection points of the long-term trend. The Monte Carlo permutation test was used for model selection, with the significance level set at *P* < 0.05. The permutation test was repeated 4499 times to compare the goodness of fit of models with different numbers of joinpoints, and the optimal model was finally determined. The Average Annual Percent Change (AAPC) and its corresponding 95% CIs were calculated based on the optimal model, and the statistical significance of the trend was determined by whether the 95% CIs of AAPC included 0.) [23]. To graphically display 204 countries or regions, R software (version 4.2.1, R Core Team, Vienna, Austria) was used for analysis, with the “ggmap” package (version 3.0.2) used for geospatial mapping, and the “ggplot2” package (version 3.4.0) used for graph drawing [26]. This package is an extension tool that makes it easy and fast to extract and apply graphic files from Google Maps. The difference between the two groups was statistically significant when the P value was less than 0.05.

## Results

### Global distribution and changing trend of DALYs caused by breast cancer

We note that there are significant geographical differences in the incidence rate of breast cancer (Table 1). Between 1990 and 2021, 85% of countries showed a growth trend. From 2019 to 2021, which is the epidemic period of COVID-19, 62% and 82% of countries will show growth trends in 2020 and 2021 respectively. Overall, DALYs show a trend of decreasing and then increasing. It is worth noting that the downward trend of San Marino was most pronounced in 2020, with DALYs decreasing from the original 150.42 to 111.07. In 2021, countries with a downward trend of over 50% include Tuvalu in Oceania, Cyprus in Western Europe, and Croatia in Central Europe. On the other hand, the country with the most significant growth trend is Turkmenistan in Central Asia, with DALYs increasing from 48.47 to 11020.31.

**Table 1:** Age-standardized rates of breast cancer-related years lived with disability (YLDs) and disability-adjusted life years (DALYs) globally and by region from 1990 to 2021. Data are presented as age-standardized rates with 95% uncertainty intervals (UIs). Values are shown for the global level, five SDI regions, and 21 GBD subregions

Global/Regions/Countries	YLDs (ASR, 95% UI)				DALYs (ASR, 95% UI)			
	1990	2019	2020	2021	1990	2019	2020	2021
Global	15.28 (11.00, 20.36)	17.15 (12.30, 22.97)	17.00 (12.06, 23.10)	17.07 (12.30, 23.27)	265.04 (250.38, 279.57)	239.96 (225.88, 254.81)	238.25 (223.77, 254.47)	239.07 (224.27, 255.05)
SDI Regions								
Low SDI	32.21 (22.97, 43.20)	31.22 (22.10, 42.46)	30.42 (21.62, 41.39)	30.29 (21.41, 41.14)	387.28 (371.79, 401.62)	252.38 (236.64, 267.28)	244.61 (228.18, 259.40)	242.88 (226.13, 257.11)
Low-middle SDI	15.02 (10.81, 20.36)	19.20 (13.74, 25.77)	19.01 (13.33, 26.14)	19.18 (13.81, 26.02)	288.36 (272.37, 303.81)	229.09 (209.79, 250.70)	224.75 (204.86, 248.23)	225.40 (205.84, 250.02)
Middle SDI	6.71 (4.81, 9.04)	12.30 (8.89, 16.31)	12.48 (8.81, 17.25)	12.69 (9.08, 17.48)	188.08 (172.67, 206.79)	214.81 (198.36, 232.54)	215.44 (197.56, 236.39)	216.98 (196.17, 239.85)
High-middle SDI	4.53 (3.20, 6.06)	8.73 (6.34, 11.57)	8.87 (6.35, 11.86)	9.00 (6.47, 12.07)	166.12 (147.76, 188.79)	246.72 (226.63, 269.97)	247.82 (225.55, 271.18)	248.83 (224.66, 273.10)
High SDI	4.78 (3.36, 6.48)	7.15 (5.18, 9.50)	7.31 (5.29, 9.87)	7.43 (5.36, 9.96)	199.33 (169.98, 232.67)	253.21 (223.27, 283.74)	256.12 (225.23, 288.60)	258.69 (229.05, 292.60)
Central Asia	12.86 (9.20, 16.95)	11.64 (8.24, 16.10)	11.47 (8.21, 15.63)	11.31 (7.98, 15.29)	340.57 (321.42, 358.52)	243.97 (226.18, 263.61)	239.07 (218.71, 260.79)	236.08 (210.17, 264.58)
Armenia	20.81 (14.48, 27.79)	19.64 (13.88, 26.98)	19.18 (13.51, 26.06)	18.76 (13.14, 25.76)	514.99 (481.02, 548.45)	332.27 (308.14, 358.64)	323.28 (294.66, 356.23)	315.26 (277.42, 362.25)
Azerbaijan	10.89 (7.66, 14.67)	12.33 (8.06, 18.27)	11.75 (7.50, 17.27)	11.67 (7.43, 16.89)	316.25 (264.05, 361.02)	262.69 (203.60, 332.29)	247.86 (187.44, 311.97)	245.58 (182.58, 316.49)
Georgia	21.81 (14.91, 29.49)	24.01 (16.79, 32.67)	23.55 (16.22, 32.58)	23.71 (16.43, 32.41)	504.45 (450.96, 558.58)	475.21 (422.80, 525.82)	463.89 (411.97, 520.31)	467.71 (399.76, 543.18)
Kazakhstan	14.63 (10.46, 19.64)	12.60 (8.54, 17.19)	12.47 (8.63, 16.97)	12.35 (8.51, 16.80)	389.41 (345.65, 433.72)	231.74 (201.85, 262.86)	227.76 (197.71, 258.96)	225.40 (188.48, 263.97)
Kyrgyzstan	11.37 (8.05, 15.55)	9.74 (6.73, 13.00)	9.75 (6.61, 13.48)	9.69 (6.55, 13.52)	321.69 (286.43, 361.43)	202.45 (175.99, 229.29)	202.10 (169.07, 235.73)	200.74 (162.79, 243.07)
Mongolia	3.02 (2.05, 4.22)	4.49 (3.00, 6.39)	4.31 (2.88, 5.96)	4.17 (2.78, 5.85)	94.62 (74.29, 119.00)	112.24 (86.92, 139.90)	106.82 (84.36, 130.64)	101.84 (78.11, 124.99)
Tajikistan	8.06 (5.50, 11.30)	6.54 (4.11, 10.36)	6.57 (4.09, 10.51)	6.65 (4.09, 10.72)	248.46 (199.42, 300.99)	177.01 (120.46, 251.23)	178.01 (116.73, 258.16)	179.90 (114.42, 265.63)
Turkmenistan	8.42 (5.80, 11.38)	9.77 (6.50, 14.79)	9.77 (6.19, 14.36)	9.71 (6.29, 14.55)	248.65 (218.00, 283.14)	232.72 (178.69, 294.66)	230.15 (172.58, 301.58)	227.26 (167.86, 303.51)
Uzbekistan	8.74 (6.03, 11.75)	8.88 (6.15, 12.18)	8.86 (6.14, 12.27)	8.75 (6.00, 12.42)	243.62 (218.87, 268.41)	209.48 (183.79, 238.31)	207.01 (177.75, 240.15)	204.97 (170.24, 245.61)
Central Europe	16.74 (12.07, 22.63)	22.91 (16.41, 30.74)	22.16 (15.72, 30.10)	22.36 (16.09, 30.17)	355.66 (341.34, 370.97)	316.17 (301.01, 330.08)	305.27 (286.62, 322.84)	308.59 (283.23, 335.65)
Albania	6.59 (4.34, 9.51)	12.29 (7.88, 17.98)	11.26 (6.87, 16.36)	11.31 (7.08, 16.63)	144.79 (113.86, 182.21)	179.94 (132.99, 234.53)	162.44 (117.68, 214.76)	162.90 (116.01, 216.44)
Bosnia and Herzegovina	10.84 (7.64, 15.04)	19.39 (13.24, 26.56)	18.40 (12.78, 25.15)	17.49 (11.69, 24.39)	232.34 (200.48, 261.92)	289.77 (252.44, 336.91)	273.49 (235.87, 319.33)	257.39 (197.86, 323.51)
Bulgaria	20.03 (14.18, 27.30)	27.72 (19.63, 38.21)	27.81 (19.47, 38.01)	28.04 (19.47, 39.41)	361.26 (322.65, 408.03)	374.12 (325.49, 428.85)	372.96 (316.09, 429.87)	376.39 (306.82, 452.81)
Croatia	22.81 (16.15, 31.79)	25.87 (18.16, 35.54)	24.78 (17.27, 34.39)	24.99 (17.60, 34.63)	381.53 (336.98, 426.25)	290.69 (258.64, 325.05)	277.05 (245.56, 310.60)	279.13 (236.60, 319.29)
Czechia	21.40 (14.96, 29.08)	23.07 (16.46, 31.83)	21.76 (15.35, 30.45)	21.87 (15.15, 30.88)	388.60 (350.95, 432.20)	251.58 (220.71, 285.13)	237.40 (204.32, 272.86)	239.81 (200.80, 281.54)
Hungary	21.43 (15.31, 28.96)	25.00 (17.46, 33.66)	24.59 (16.71, 33.40)	24.68 (17.05, 34.86)	439.87 (390.13, 496.08)	323.02 (285.51, 361.09)	316.47 (273.64, 359.53)	315.75 (266.15, 369.72)
Montenegro	24.59 (15.95, 36.30)	34.81 (23.91, 49.76)	33.93 (23.28, 48.22)	31.38 (21.35, 43.90)	380.72 (296.18, 490.22)	442.52 (346.45, 558.06)	429.85 (334.66, 537.08)	397.36 (308.55, 500.14)
North Macedonia	16.92 (12.02, 23.33)	24.07 (16.91, 33.14)	23.24 (16.08, 32.13)	23.22 (16.03, 33.48)	386.01 (317.77, 462.17)	375.20 (306.32, 450.07)	360.87 (291.69, 442.43)	361.48 (277.16, 459.97)
Poland	14.95 (10.73, 20.25)	22.45 (15.99, 30.00)	21.44 (14.95, 28.74)	21.72 (15.36, 28.92)	346.57 (334.28, 358.30)	318.13 (301.55, 332.73)	303.39 (281.25, 325.80)	307.96 (271.36, 344.40)
Romania	12.60 (8.86, 17.23)	19.63 (14.26, 26.81)	19.31 (13.68, 26.91)	20.02 (13.94, 27.63)	309.88 (289.97, 332.79)	314.04 (289.51, 337.62)	307.49 (276.17, 338.18)	320.84 (279.15, 361.92)
Serbia	19.03 (13.05, 26.49)	27.15 (18.57, 37.94)	27.05 (18.32, 38.32)	26.97 (18.70, 38.96)	436.98 (332.33, 552.46)	403.86 (324.92, 484.76)	400.89 (312.19, 494.40)	398.80 (301.87, 507.47)
Slovakia	17.14 (12.10, 23.50)	23.09 (15.26, 31.78)	22.44 (15.07, 31.28)	22.45 (15.11, 32.37)	356.05 (310.33, 412.32)	304.91 (250.97, 369.24)	295.97 (238.06, 359.35)	297.85 (234.14, 368.73)
Slovenia	21.83 (15.37, 29.69)	24.61 (17.15, 34.18)	22.33 (15.38, 30.71)	21.88 (15.26, 30.74)	366.00 (335.24, 400.03)	249.92 (219.93, 281.73)	228.72 (199.32, 261.83)	221.84 (182.66, 266.31)
Eastern Europe	16.38 (11.81, 21.86)	20.23 (14.44, 27.22)	20.01 (14.44, 26.82)	20.44 (14.98, 27.10)	347.02 (336.12, 357.75)	304.52 (286.12, 324.99)	298.20 (277.85, 321.07)	305.14 (271.86, 344.55)
Belarus	16.48 (11.91, 22.75)	20.26 (14.10, 27.57)	20.41 (14.09, 28.95)	20.55 (14.40, 29.72)	319.92 (288.93, 353.35)	257.64 (228.58, 291.24)	258.55 (215.07, 310.10)	259.86 (201.99, 328.51)
Estonia	20.05 (14.35, 26.65)	22.01 (15.35, 30.73)	21.85 (15.19, 30.03)	21.25 (14.52, 29.86)	373.68 (342.19, 407.43)	249.76 (217.01, 281.41)	249.16 (216.97, 284.79)	239.84 (198.34, 284.26)
Latvia	18.34 (13.01, 24.62)	21.11 (14.80, 29.47)	20.44 (14.10, 28.45)	20.76 (14.17, 29.66)	369.20 (327.26, 419.54)	301.57 (260.47, 346.55)	290.50 (245.26, 337.46)	295.10 (240.82, 351.27)
Lithuania	18.80 (13.26, 24.97)	20.83 (14.53, 28.39)	20.59 (14.36, 28.08)	20.95 (14.54, 29.36)	333.27 (310.47, 357.98)	287.10 (254.85, 319.51)	280.84 (244.97, 317.40)	285.99 (241.35, 333.52)
Republic of Moldova	16.66 (11.81, 22.61)	18.10 (12.57, 25.21)	18.51 (13.02, 25.52)	18.73 (12.86, 26.09)	390.77 (346.82, 434.28)	292.69 (248.89, 340.56)	297.37 (256.60, 345.44)	298.96 (252.07, 358.62)
Russian Federation	15.45 (11.06, 20.66)	21.80 (15.61, 29.12)	21.75 (15.58, 28.94)	22.34 (15.98, 29.97)	310.72 (302.10, 319.26)	303.69 (292.64, 313.58)	300.95 (283.50, 318.21)	309.59 (273.51, 343.36)
Ukraine	18.51 (13.12, 24.81)	15.38 (9.98, 21.85)	14.53 (9.60, 20.32)	14.56 (8.70, 21.88)	445.85 (415.09, 477.70)	318.70 (243.74, 401.54)	298.59 (232.68, 384.51)	302.22 (189.57, 445.61)
Australasia	32.94 (23.49, 44.40)	34.01 (23.92, 46.64)	32.23 (22.72, 44.12)	32.53 (22.94, 44.61)	422.78 (399.83, 444.90)	257.15 (234.23, 279.99)	242.08 (218.20, 265.45)	244.57 (220.14, 271.18)
Australia	31.07 (22.26, 41.92)	33.58 (23.51, 45.94)	31.77 (22.35, 43.42)	31.93 (22.22, 43.75)	400.65 (378.10, 424.48)	249.00 (224.77, 273.84)	234.08 (208.69, 259.65)	235.60 (209.33, 262.50)
New Zealand	42.39 (30.39, 57.40)	36.22 (25.47, 49.57)	34.58 (24.23, 46.96)	35.64 (25.02, 48.54)	535.25 (499.88, 574.71)	298.32 (272.67, 323.95)	282.42 (257.42, 306.75)	290.52 (263.68, 317.66)
High-income Asia Pacific	13.02 (9.05, 17.76)	22.92 (16.10, 31.17)	22.57 (15.82, 30.96)	22.42 (15.71, 30.81)	137.49 (131.64, 143.86)	168.19 (155.27, 179.38)	164.73 (151.84, 176.60)	163.10 (149.52, 175.58)
Brunei Darussalam	11.91 (7.43, 17.04)	19.01 (12.62, 25.86)	18.57 (12.31, 25.54)	17.82 (11.61, 24.76)	274.18 (203.44, 364.45)	333.52 (271.73, 405.14)	324.56 (255.04, 395.16)	304.97 (236.64, 374.51)
Japan	14.42 (9.94, 19.65)	25.66 (18.03, 35.16)	25.31 (17.84, 34.77)	25.17 (17.64, 34.60)	142.51 (136.17, 148.35)	185.68 (171.93, 197.79)	182.18 (168.58, 194.22)	180.42 (166.34, 192.11)
Republic of Korea	6.44 (4.37, 9.24)	15.38 (10.31, 21.72)	15.31 (10.06, 21.63)	15.37 (10.29, 21.65)	103.65 (91.44, 122.79)	119.76 (100.57, 137.96)	118.15 (96.82, 139.09)	118.44 (96.51, 140.42)
Singapore	16.76 (11.90, 22.88)	23.89 (16.88, 33.38)	22.85 (16.05, 31.80)	22.09 (15.49, 30.51)	258.07 (241.56, 275.15)	192.03 (178.20, 208.51)	181.68 (167.74, 197.40)	174.42 (159.84, 190.41)
High-income North America	49.18 (35.06, 65.59)	38.25 (27.13, 51.32)	37.77 (26.95, 50.72)	37.92 (27.03, 50.77)	479.56 (457.57, 500.81)	277.30 (260.57, 293.67)	273.24 (255.74, 290.37)	273.50 (256.52, 290.08)
Canada	39.55 (27.94, 52.81)	32.67 (22.50, 43.83)	30.99 (21.60, 41.77)	30.95 (21.94, 41.59)	430.96 (401.19, 460.51)	256.26 (231.79, 280.33)	242.40 (218.15, 267.37)	241.05 (217.24, 266.74)
Greenland	19.13 (13.23, 26.56)	15.23 (10.32, 22.71)	15.95 (10.65, 23.66)	15.04 (9.84, 22.16)	466.52 (373.26, 580.18)	260.45 (200.37, 332.60)	272.49 (206.57, 347.30)	253.99 (189.42, 332.84)
United States of America	50.20 (35.87, 67.15)	38.92 (27.63, 52.16)	38.59 (27.51, 51.67)	38.77 (27.63, 51.97)	485.06 (462.75, 506.98)	279.77 (263.47, 296.80)	276.91 (260.06, 294.68)	277.38 (260.38, 295.21)
Southern Latin America	17.67 (12.65, 24.04)	20.54 (14.39, 27.86)	19.93 (14.07, 27.14)	19.29 (13.65, 26.56)	441.05 (417.90, 460.93)	346.20 (321.16, 368.50)	331.73 (307.44, 353.61)	317.65 (292.89, 341.57)
Argentina	18.82 (13.58, 25.66)	22.15 (15.54, 29.97)	21.73 (15.21, 29.58)	20.58 (14.41, 28.12)	480.92 (452.86, 505.58)	398.50 (367.89, 428.05)	385.48 (355.54, 412.75)	361.83 (331.24, 391.64)
Chile	11.64 (8.26, 15.88)	15.28 (10.68, 21.08)	14.41 (10.13, 19.78)	14.63 (10.20, 20.66)	274.66 (257.31, 292.63)	207.41 (189.56, 224.26)	192.95 (175.85, 210.57)	194.60 (176.70, 213.16)
Uruguay	24.96 (17.50, 33.71)	29.02 (20.46, 38.92)	27.92 (19.65, 37.66)	28.88 (20.36, 38.99)	579.08 (546.75, 610.16)	464.68 (428.90, 498.35)	441.02 (404.40, 474.69)	456.31 (416.96, 492.60)
Western Europe	33.84 (24.06, 45.59)	35.09 (24.84, 47.88)	33.29 (23.46, 45.38)	33.13 (23.33, 44.89)	472.38 (454.13, 489.00)	292.73 (271.48, 311.84)	276.29 (254.72, 295.18)	274.36 (252.48, 293.44)
Andorra	28.80 (17.63, 44.16)	39.25 (25.34, 58.30)	32.81 (20.64, 50.13)	32.55 (20.43, 50.63)	354.39 (250.31, 510.00)	324.52 (236.15, 428.25)	264.68 (179.90, 363.88)	262.34 (177.37, 367.67)
Austria	32.29 (22.98, 43.50)	28.68 (19.92, 39.06)	27.02 (18.84, 36.94)	26.11 (18.05, 35.92)	451.97 (422.86, 480.67)	265.62 (242.07, 291.60)	249.62 (226.92, 273.25)	239.18 (216.40, 262.44)
Belgium	40.50 (28.54, 54.18)	34.76 (24.19, 47.74)	31.61 (21.94, 43.36)	33.33 (23.23, 45.71)	560.50 (525.04, 596.01)	308.37 (280.89, 334.54)	277.93 (250.83, 303.33)	291.96 (263.65, 318.23)
Cyprus	25.12 (17.17, 35.37)	40.17 (26.90, 55.88)	37.91 (25.60, 52.96)	37.20 (24.96, 52.27)	369.79 (308.23, 444.08)	326.32 (272.93, 392.26)	304.98 (249.22, 364.51)	297.34 (244.89, 357.91)
Denmark	36.83 (26.38, 48.83)	30.24 (21.21, 40.84)	29.71 (20.47, 40.85)	28.75 (20.00, 39.31)	631.35 (598.20, 666.14)	284.97 (258.23, 311.14)	278.69 (252.97, 305.08)	269.27 (243.69, 297.19)
Finland	31.00 (21.86, 41.99)	34.00 (23.72, 47.16)	33.13 (23.18, 46.34)	32.44 (22.68, 44.64)	382.22 (358.97, 406.87)	245.29 (220.75, 271.59)	238.21 (215.04, 263.28)	233.13 (209.01, 257.98)
France	32.75 (22.84, 45.39)	43.43 (30.33, 60.22)	42.19 (29.13, 58.80)	40.56 (27.95, 56.12)	448.46 (424.77, 474.75)	325.76 (295.64, 355.56)	315.19 (284.15, 345.85)	301.59 (269.46, 331.41)
Germany	31.66 (22.58, 43.34)	35.76 (25.24, 49.29)	34.52 (24.31, 47.33)	33.90 (23.98, 46.06)	471.13 (442.31, 499.58)	312.60 (288.71, 336.61)	300.79 (276.44, 324.97)	294.19 (268.88, 319.37)
Greece	33.16 (23.37, 45.00)	32.95 (22.86, 44.88)	33.30 (23.88, 45.96)	33.34 (23.23, 46.39)	406.32 (382.43, 429.00)	314.24 (291.63, 337.49)	317.67 (292.31, 343.04)	318.22 (292.50, 344.83)
Iceland	35.60 (25.23, 48.22)	30.66 (21.43, 42.81)	30.48 (21.40, 42.68)	32.01 (22.22, 44.80)	422.22 (387.11, 455.37)	243.78 (216.68, 272.70)	239.81 (212.59, 268.95)	253.09 (222.79, 284.34)
Ireland	36.10 (25.75, 48.83)	37.73 (26.51, 51.03)	34.90 (24.84, 47.76)	33.61 (23.65, 46.09)	525.28 (496.10, 559.15)	304.49 (274.72, 336.15)	278.91 (251.79, 310.19)	266.31 (238.72, 296.34)
Israel	30.32 (21.41, 41.57)	30.83 (21.18, 42.67)	28.65 (20.12, 39.53)	27.97 (19.51, 39.45)	486.15 (456.47, 516.80)	302.77 (272.44, 331.21)	278.32 (248.44, 307.13)	269.95 (240.49, 297.86)
Italy	36.48 (25.96, 49.61)	36.07 (25.51, 49.12)	33.35 (23.19, 45.44)	34.30 (24.26, 46.68)	459.77 (439.20, 477.85)	290.75 (265.97, 311.28)	268.60 (244.61, 288.89)	275.53 (250.69, 296.43)
Luxembourg	34.64 (24.46, 46.43)	31.53 (22.44, 42.96)	28.42 (20.19, 38.97)	28.85 (20.44, 39.17)	520.72 (487.91, 553.55)	275.77 (250.97, 302.89)	244.98 (220.72, 270.33)	247.20 (221.25, 275.16)
Malta	33.08 (23.35, 45.36)	30.70 (21.69, 42.55)	28.76 (20.24, 40.00)	31.23 (21.86, 43.83)	536.94 (498.71, 580.43)	302.02 (272.41, 336.49)	279.62 (249.31, 312.14)	304.60 (271.18, 341.16)
Monaco	45.51 (29.83, 65.75)	65.31 (42.77, 97.50)	64.76 (43.14, 97.84)	64.76 (43.12, 98.73)	537.04 (398.95, 704.76)	563.83 (420.24, 748.84)	557.70 (416.41, 748.59)	556.81 (413.56, 753.58)
Netherlands	36.47 (25.63, 49.26)	37.59 (26.78, 52.27)	35.71 (25.08, 49.39)	35.36 (24.59, 49.01)	532.41 (499.34, 566.58)	318.15 (292.09, 343.86)	301.27 (275.93, 328.36)	299.26 (273.77, 327.51)
Norway	26.08 (18.68, 35.17)	25.34 (17.99, 34.98)	24.75 (17.54, 33.57)	24.19 (16.96, 32.85)	372.62 (355.73, 389.95)	204.96 (189.15, 219.82)	199.01 (182.52, 214.43)	194.64 (178.26, 209.74)
Portugal	26.62 (18.84, 35.96)	31.27 (21.87, 43.45)	29.50 (20.59, 41.28)	29.83 (20.85, 40.98)	421.41 (396.59, 449.32)	272.03 (247.31, 295.36)	254.33 (230.63, 276.02)	255.84 (231.25, 278.79)
San Marino	27.31 (18.82, 38.47)	30.79 (19.82, 45.29)	23.16 (13.74, 36.55)	22.11 (12.51, 35.97)	301.87 (238.20, 379.56)	256.88 (188.76, 340.53)	186.57 (120.08, 268.28)	176.42 (104.70, 261.31)
Spain	27.53 (19.33, 37.24)	27.25 (18.80, 37.76)	25.26 (17.50, 35.03)	25.91 (17.72, 36.14)	397.58 (372.48, 422.63)	227.08 (206.59, 248.81)	208.74 (187.69, 229.04)	213.26 (192.91, 234.42)
Sweden	31.19 (21.89, 43.02)	28.56 (20.16, 39.00)	26.48 (18.45, 36.05)	25.99 (17.97, 36.24)	355.14 (330.99, 380.70)	216.31 (197.11, 235.96)	199.13 (180.67, 219.02)	194.66 (164.42, 223.82)
Switzerland	30.61 (21.74, 40.98)	28.39 (19.87, 38.25)	25.94 (18.10, 35.47)	25.95 (18.01, 35.81)	396.77 (370.23, 422.66)	229.27 (207.09, 251.10)	207.67 (184.25, 227.79)	207.67 (184.28, 227.79)
United Kingdom	40.96 (29.35, 55.00)	35.30 (25.11, 47.82)	33.20 (23.48, 44.93)	33.44 (23.81, 45.35)	577.81 (559.32, 594.44)	305.66 (287.51, 320.55)	285.05 (269.00, 300.35)	286.30 (269.27, 301.45)
Andean Latin America	5.75 (4.04, 7.77)	10.66 (7.39, 14.75)	10.22 (7.12, 14.38)	10.31 (7.01, 14.90)	195.24 (166.48, 227.20)	216.12 (180.62, 262.80)	206.38 (166.86, 254.00)	206.64 (163.49, 261.01)
Bolivia (Plurinational State of)	6.31 (3.67, 9.89)	9.82 (6.09, 15.17)	9.86 (6.06, 15.53)	10.00 (6.21, 15.72)	254.23 (166.67, 367.28)	278.85 (189.70, 400.50)	279.60 (187.81, 404.00)	280.72 (185.91, 410.27)
Ecuador	4.53 (3.24, 6.21)	9.96 (6.73, 13.62)	9.42 (6.35, 13.14)	9.57 (6.26, 13.81)	144.52 (135.25, 153.70)	211.83 (184.07, 243.22)	198.72 (159.92, 243.85)	200.32 (152.02, 256.03)
Peru	6.14 (4.25, 8.33)	11.27 (7.28, 16.26)	10.72 (7.13, 15.61)	10.77 (6.87, 16.25)	201.80 (165.32, 239.63)	200.55 (154.16, 256.21)	189.42 (139.16, 245.84)	188.92 (136.75, 249.00)
Caribbean	13.43 (9.75, 18.08)	17.34 (12.45, 23.28)	17.02 (12.01, 23.17)	17.06 (12.11, 23.08)	304.93 (278.75, 336.34)	322.32 (275.71, 375.99)	314.95 (267.52, 373.38)	314.15 (262.03, 374.18)
Antigua and Barbuda	19.04 (13.73, 25.58)	30.42 (21.98, 41.02)	27.49 (19.64, 37.11)	27.53 (20.08, 36.11)	420.14 (378.81, 464.89)	523.22 (460.93, 592.47)	471.93 (412.56, 536.10)	462.49 (431.52, 499.73)
Bahamas	24.28 (17.43, 32.41)	31.86 (21.98, 44.10)	31.45 (21.44, 44.42)	31.60 (21.24, 44.73)	606.57 (553.65, 662.38)	635.82 (516.58, 760.50)	628.49 (502.07, 766.53)	626.92 (503.01, 785.96)
Barbados	24.19 (17.36, 32.38)	35.02 (24.90, 48.65)	34.05 (24.08, 47.55)	32.86 (22.75, 47.37)	522.76 (479.64, 564.82)	583.35 (478.41, 705.02)	563.14 (458.29, 696.42)	538.30 (428.82, 682.06)
Belize	5.90 (4.12, 8.09)	10.22 (7.24, 13.97)	9.62 (6.95, 12.77)	9.77 (7.10, 13.13)	148.09 (134.36, 161.46)	225.94 (201.06, 252.10)	210.44 (189.45, 233.66)	212.25 (186.84, 241.52)
Bermuda	31.48 (22.94, 42.84)	31.02 (21.65, 44.58)	29.47 (20.64, 42.66)	31.19 (21.63, 45.30)	551.05 (495.99, 614.57)	304.62 (254.18, 371.04)	286.90 (240.54, 352.59)	303.94 (250.69, 382.03)
Cuba	15.89 (11.47, 21.31)	20.09 (14.35, 27.24)	19.71 (14.01, 27.02)	19.75 (13.78, 28.23)	296.14 (275.71, 315.16)	272.03 (251.70, 294.31)	264.22 (241.25, 289.91)	262.38 (223.25, 310.39)
Dominica	17.22 (12.17, 23.65)	19.56 (13.72, 27.86)	19.60 (13.55, 27.67)	19.57 (13.50, 27.71)	445.54 (381.45, 510.84)	455.97 (360.80, 562.70)	453.60 (351.07, 561.90)	451.23 (341.10, 565.35)
Dominican Republic	6.35 (4.46, 8.78)	9.93 (6.63, 14.48)	9.66 (6.40, 13.55)	9.64 (6.32, 13.98)	187.74 (158.20, 220.15)	234.22 (183.72, 292.95)	222.80 (170.16, 278.19)	220.31 (167.10, 290.78)
Grenada	16.22 (11.82, 21.58)	22.83 (16.14, 30.54)	22.96 (16.51, 30.99)	22.78 (16.10, 31.25)	452.38 (405.16, 504.39)	482.69 (432.25, 536.05)	479.32 (424.98, 540.36)	471.94 (407.83, 537.24)
Guyana	9.68 (6.87, 13.31)	14.48 (9.67, 20.22)	14.67 (9.94, 20.92)	14.74 (10.12, 20.94)	328.87 (286.99, 377.33)	429.25 (334.97, 544.30)	426.41 (326.13, 548.55)	422.26 (316.57, 551.98)
Haiti	8.32 (4.67, 13.91)	10.90 (6.22, 17.74)	10.85 (6.24, 17.62)	10.90 (6.22, 17.39)	355.94 (216.60, 551.42)	414.37 (255.21, 640.91)	411.95 (253.44, 643.13)	411.02 (253.64, 642.60)
Jamaica	14.27 (10.08, 19.10)	24.32 (16.73, 35.50)	23.80 (16.39, 34.94)	24.22 (16.53, 35.69)	315.49 (291.87, 341.50)	454.71 (347.20, 572.18)	441.80 (326.62, 578.33)	448.12 (336.95, 582.46)
Puerto Rico	17.41 (12.22, 23.18)	23.11 (16.63, 30.65)	22.90 (16.33, 31.18)	23.07 (16.30, 32.36)	319.61 (296.71, 341.24)	271.14 (249.96, 293.65)	264.79 (238.96, 288.04)	264.34 (217.74, 312.27)
Saint Kitts and Nevis	21.83 (15.73, 28.81)	20.80 (14.78, 28.48)	20.88 (14.65, 29.11)	21.08 (15.14, 29.37)	681.16 (620.45, 747.12)	420.47 (355.56, 494.12)	418.13 (345.92, 495.96)	420.52 (343.63, 506.34)
Saint Lucia	17.67 (12.90, 23.35)	17.12 (12.01, 23.20)	17.54 (12.15, 24.29)	18.01 (12.68, 25.41)	488.71 (451.60, 527.12)	348.44 (296.35, 412.89)	355.43 (298.99, 429.06)	362.28 (301.32, 443.59)
Saint Vincent and the Grenadines	17.80 (12.92, 23.92)	19.93 (14.05, 27.93)	20.04 (14.09, 27.90)	20.30 (14.68, 28.06)	481.46 (431.50, 525.83)	456.64 (406.30, 512.56)	454.95 (401.41, 522.82)	456.79 (394.84, 532.15)
Suriname	8.96 (6.15, 11.92)	12.44 (8.60, 17.27)	12.30 (8.55, 17.09)	11.53 (7.84, 16.83)	267.48 (225.12, 312.12)	315.90 (269.53, 367.98)	310.91 (258.93, 369.66)	290.24 (221.15, 372.70)
Trinidad and Tobago	15.76 (11.37, 21.16)	20.73 (14.61, 28.48)	20.86 (14.30, 29.48)	20.86 (14.11, 30.06)	422.02 (390.44, 457.78)	415.75 (350.97, 483.50)	416.84 (330.53, 507.74)	414.49 (314.81, 536.92)
United States Virgin Islands	21.51 (14.79, 29.50)	24.91 (16.07, 39.18)	22.05 (13.93, 35.12)	20.41 (12.94, 32.17)	480.52 (402.01, 574.91)	435.16 (308.69, 615.41)	385.81 (269.15, 554.31)	350.31 (244.65, 495.38)
Central Latin America	9.03 (6.60, 11.87)	16.88 (12.37, 22.95)	17.17 (12.19, 23.21)	17.54 (12.59, 24.03)	195.63 (189.88, 201.64)	236.38 (224.53, 249.48)	238.30 (216.90, 259.58)	241.71 (209.04, 273.49)
Colombia	10.89 (7.72, 14.58)	20.41 (14.75, 27.86)	20.87 (14.65, 28.17)	21.05 (14.51, 29.32)	231.24 (216.42, 247.81)	243.33 (223.43, 263.97)	245.42 (220.84, 268.77)	244.68 (203.76, 295.82)
Costa Rica	13.51 (9.33, 17.89)	24.75 (17.69, 33.66)	24.85 (17.70, 33.80)	26.56 (18.92, 36.74)	199.82 (184.81, 214.80)	263.08 (241.68, 284.01)	260.69 (238.97, 285.87)	276.90 (243.05, 315.74)
EI Salvador	5.66 (3.94, 7.81)	15.87 (10.83, 21.30)	16.13 (10.71, 22.57)	16.31 (10.90, 23.34)	137.68 (119.37, 158.00)	221.53 (191.64, 253.55)	223.03 (178.65, 269.38)	223.10 (176.34, 284.07)
Guatemala	3.31 (2.38, 4.39)	8.02 (5.77, 10.80)	7.70 (5.32, 10.39)	7.80 (5.36, 10.72)	94.71 (88.59, 100.58)	160.52 (150.08, 172.80)	151.26 (136.78, 164.98)	151.82 (127.35, 178.46)
Honduras	4.78 (2.98, 7.03)	9.73 (6.16, 14.84)	9.92 (6.23, 15.17)	10.06 (6.53, 15.10)	131.69 (95.58, 169.64)	211.86 (150.92, 297.63)	213.48 (150.90, 300.46)	214.83 (153.23, 295.62)
Mexico	8.64 (6.31, 11.36)	15.17 (10.94, 20.23)	15.50 (11.00, 20.97)	15.99 (11.17, 22.35)	190.70 (184.82, 195.95)	223.98 (216.79, 231.80)	226.67 (200.04, 254.81)	231.73 (192.21, 275.22)
Nicaragua	4.84 (3.27, 6.53)	11.06 (7.88, 15.22)	10.35 (7.26, 14.60)	10.38 (7.04, 15.41)	106.60 (91.10, 123.85)	166.39 (146.52, 188.09)	153.69 (131.42, 179.30)	153.15 (119.17, 193.39)
Panama	10.56 (7.55, 14.33)	21.04 (15.25, 27.78)	22.25 (15.87, 30.01)	22.26 (15.41, 31.95)	179.35 (165.80, 193.32)	235.76 (215.36, 254.39)	246.35 (221.65, 270.95)	241.95 (191.53, 293.70)
Venezuela (Bolivarian Republic of)	11.02 (7.93, 14.57)	22.31 (15.57, 32.43)	22.63 (15.45, 33.04)	23.01 (15.53, 33.17)	235.81 (221.88, 249.99)	322.16 (258.15, 393.57)	329.21 (255.36, 420.79)	335.69 (256.79, 431.42)
Tropical Latin America	9.00 (6.56, 11.78)	13.28 (9.64, 17.52)	13.54 (9.79, 17.82)	13.93 (10.08, 18.31)	268.48 (258.43, 278.26)	277.78 (264.94, 288.75)	281.23 (266.64, 293.88)	287.77 (269.86, 302.75)
Brazil	9.05 (6.60, 11.83)	13.28 (9.66, 17.51)	13.56 (9.81, 17.80)	13.96 (10.11, 18.33)	270.17 (260.15, 280.00)	277.56 (264.85, 288.47)	281.44 (266.67, 294.05)	288.09 (270.87, 303.84)
Paraguay	7.07 (4.87, 9.87)	13.31 (8.80, 18.49)	12.91 (8.68, 18.27)	13.08 (8.36, 19.12)	199.03 (161.11, 240.74)	289.49 (229.48, 356.89)	275.57 (216.14, 349.86)	278.11 (206.64, 370.82)
North Africa and Middle East	6.04 (4.31, 8.11)	15.93 (11.55, 21.67)	15.88 (11.41, 21.65)	16.13 (11.51, 22.00)	124.15 (110.45, 142.12)	196.43 (179.54, 217.09)	194.28 (172.41, 219.35)	194.87 (170.24, 224.24)
Afghanistan	4.93 (2.42, 8.78)	8.57 (4.26, 15.86)	8.76 (4.35, 16.31)	9.02 (4.47, 16.85)	163.36 (81.16, 286.06)	238.79 (126.09, 410.96)	241.75 (127.48, 417.68)	244.78 (128.56, 425.51)
Algeria	5.72 (3.95, 8.23)	9.77 (6.57, 14.26)	9.91 (6.60, 14.66)	10.10 (6.74, 15.34)	114.86 (87.24, 147.96)	127.60 (97.01, 164.42)	128.10 (97.23, 165.15)	128.95 (97.23, 169.08)
Bahrain	16.76 (11.89, 22.63)	30.34 (20.95, 42.67)	30.48 (20.63, 43.43)	30.42 (20.78, 43.27)	321.62 (269.95, 376.56)	290.76 (226.36, 367.79)	290.06 (225.76, 370.60)	287.31 (224.70, 366.74)
Egypt	6.36 (4.29, 8.86)	17.23 (11.96, 23.15)	17.66 (12.48, 24.14)	18.18 (12.38, 25.73)	148.84 (128.45, 177.79)	263.91 (235.64, 296.42)	265.94 (219.06, 312.99)	268.96 (213.47, 332.92)
Iran (Islamic Republic of)	7.39 (5.04, 10.19)	19.55 (13.77, 26.50)	18.18 (12.97, 24.67)	18.35 (12.98, 25.05)	111.01 (95.67, 127.87)	179.29 (161.62, 198.13)	164.57 (149.50, 180.78)	164.51 (147.41, 183.94)
Iraq	9.22 (6.06, 13.18)	19.82 (12.91, 30.06)	20.44 (13.65, 30.91)	20.87 (13.56, 31.48)	205.84 (152.11, 269.45)	271.78 (198.53, 359.94)	275.33 (199.21, 364.00)	275.70 (197.01, 367.27)
Jordan	11.79 (7.77, 16.80)	21.74 (13.77, 32.50)	21.98 (14.19, 32.94)	22.08 (14.05, 32.75)	230.69 (176.11, 297.90)	234.15 (167.45, 311.13)	234.42 (167.31, 312.25)	233.62 (167.75, 312.66)
Kuwait	11.36 (8.19, 15.62)	17.99 (12.51, 24.84)	17.53 (12.30, 24.16)	18.80 (13.24, 25.63)	140.82 (125.33, 155.90)	135.93 (120.22, 151.52)	131.50 (115.44, 148.27)	141.24 (117.38, 163.58)
Lebanon	16.30 (10.14, 23.81)	35.08 (24.05, 49.91)	35.54 (24.07, 49.95)	35.83 (24.22, 50.91)	299.14 (213.54, 403.67)	332.57 (273.65, 405.41)	333.91 (268.69, 408.74)	334.74 (267.91, 410.71)
Libya	7.01 (4.58, 10.02)	16.69 (10.71, 24.79)	16.35 (10.46, 24.21)	16.06 (10.14, 24.28)	128.56 (101.64, 166.65)	208.94 (155.11, 271.80)	209.09 (157.12, 283.10)	209.29 (154.77, 288.73)
Morocco	4.19 (2.78, 6.16)	9.34 (5.57, 14.30)	9.60 (5.65, 14.79)	9.80 (5.70, 15.28)	98.65 (75.83, 128.89)	161.45 (109.83, 230.45)	162.80 (107.84, 236.11)	163.82 (108.73, 237.29)
Oman	3.40 (2.16, 4.84)	7.03 (4.77, 9.91)	6.50 (4.34, 9.01)	6.46 (4.41, 9.23)	56.12 (40.73, 75.56)	69.30 (55.68, 85.85)	62.63 (48.97, 78.04)	61.61 (47.14, 78.32)
Palestine	14.64 (9.05, 21.54)	25.46 (17.61, 34.58)	25.32 (17.41, 34.87)	26.30 (17.86, 36.26)	304.45 (216.44, 417.21)	330.62 (275.87, 388.73)	322.87 (261.70, 388.30)	331.53 (267.66, 405.50)
Qatar	13.81 (9.53, 18.78)	28.19 (18.99, 41.47)	27.36 (18.70, 39.94)	28.51 (19.10, 41.33)	232.02 (183.72, 281.80)	221.99 (170.11, 289.31)	211.25 (160.21, 279.27)	216.90 (161.59, 290.31)
Saudi Arabia	4.82 (3.25, 6.93)	12.71 (8.30, 18.06)	12.74 (8.28, 18.36)	12.85 (8.38, 18.76)	92.33 (67.35, 124.92)	142.39 (106.66, 190.21)	140.99 (105.15, 192.44)	139.85 (102.81, 188.24)
Sudan	3.48 (1.98, 5.59)	6.55 (3.94, 10.81)	6.78 (4.06, 11.26)	7.04 (4.11, 12.01)	91.75 (57.26, 140.87)	129.68 (79.07, 198.92)	132.17 (80.65, 205.28)	134.38 (81.64, 213.96)
Syrian Arab Republic	7.25 (4.72, 10.37)	15.28 (9.78, 23.48)	15.50 (9.95, 23.72)	15.81 (10.18, 23.85)	147.03 (109.75, 185.63)	184.13 (139.05, 246.90)	184.80 (137.10, 246.79)	185.69 (136.55, 249.05)
Tunisia	8.33 (5.55, 12.09)	17.00 (11.39, 26.48)	17.01 (11.39, 26.43)	17.21 (11.50, 26.56)	150.18 (122.90, 182.63)	186.97 (131.75, 257.06)	185.18 (131.49, 257.81)	186.85 (133.43, 255.21)
Turkey	4.70 (3.21, 6.68)	19.94 (13.71, 28.99)	20.22 (13.27, 29.42)	20.50 (13.29, 29.80)	96.17 (77.21, 117.43)	214.37 (179.16, 254.36)	214.64 (171.17, 261.44)	214.05 (168.63, 265.16)
United Arab Emirates	11.61 (7.78, 16.81)	25.03 (16.84, 36.10)	20.20 (13.13, 29.74)	18.92 (12.54, 28.07)	235.56 (177.53, 307.06)	297.64 (222.34, 386.43)	236.57 (173.82, 309.64)	220.33 (163.01, 286.11)
Yemen	3.08 (1.90, 4.72)	5.72 (3.53, 9.47)	5.86 (3.79, 9.42)	5.98 (3.75, 9.79)	76.86 (48.48, 107.30)	124.49 (87.22, 173.94)	127.25 (87.76, 181.55)	128.92 (88.26, 184.39)
South Asia	3.85 (2.75, 5.13)	7.46 (5.20, 9.87)	7.60 (5.26, 10.06)	7.72 (5.31, 10.59)	152.41 (134.77, 171.94)	221.83 (198.63, 246.48)	222.64 (197.67, 248.77)	223.52 (195.29, 255.51)
Bangladesh	2.54 (1.62, 3.71)	5.14 (3.43, 7.66)	5.33 (3.50, 7.85)	5.52 (3.57, 7.98)	108.20 (80.61, 147.00)	142.41 (109.69, 180.39)	145.51 (111.38, 184.51)	148.02 (113.00, 189.10)
Bhutan	2.98 (1.91, 4.43)	4.72 (2.99, 7.20)	4.80 (3.12, 7.38)	4.86 (3.10, 7.36)	119.73 (84.32, 158.16)	132.26 (91.16, 181.40)	132.87 (91.79, 183.84)	132.93 (92.42, 183.71)
India	3.62 (2.54, 4.89)	7.21 (5.01, 9.61)	7.33 (5.01, 9.83)	7.44 (5.00, 10.38)	140.62 (119.40, 163.89)	205.81 (180.05, 236.63)	205.81 (177.99, 237.59)	206.15 (174.52, 243.66)
Nepal	2.95 (1.92, 4.23)	4.93 (3.06, 7.39)	5.07 (3.14, 7.82)	5.17 (3.25, 7.96)	122.70 (89.92, 160.29)	150.65 (108.10, 206.45)	152.63 (109.48, 210.42)	153.84 (111.35, 210.49)
Pakistan	7.06 (4.72, 10.05)	12.45 (8.33, 17.59)	12.71 (8.43, 17.92)	12.92 (8.40, 18.31)	297.52 (234.25, 369.19)	449.67 (328.77, 590.63)	454.51 (336.15, 607.78)	457.43 (326.92, 606.57)
East Asia	6.33 (4.38, 8.83)	13.08 (8.91, 18.16)	13.43 (8.89, 19.77)	13.62 (9.09, 19.33)	152.24 (125.70, 184.15)	146.57 (117.75, 181.37)	148.41 (116.76, 187.90)	148.72 (116.72, 187.14)
China	6.30 (4.33, 8.80)	13.00 (8.82, 18.19)	13.38 (8.79, 19.84)	13.58 (9.05, 19.36)	151.48 (123.80, 184.38)	143.86 (113.72, 180.39)	145.92 (112.64, 187.27)	146.29 (113.15, 186.04)
Democratic People’s Republic of Korea	6.16 (3.78, 9.25)	8.84 (5.34, 13.22)	8.83 (5.31, 13.14)	8.84 (5.45, 13.55)	183.62 (121.97, 269.37)	203.92 (143.28, 275.88)	202.59 (140.24, 276.83)	201.06 (137.43, 274.48)
Taiwan (Province of China)	8.31 (5.76, 11.62)	20.96 (14.81, 28.51)	20.51 (14.17, 27.83)	20.44 (14.49, 27.42)	160.16 (150.37, 169.33)	242.06 (225.26, 258.77)	234.77 (217.69, 250.43)	232.77 (212.88, 251.83)
Oceania	8.82 (6.03, 12.40)	9.93 (7.02, 14.10)	9.90 (7.16, 14.16)	9.85 (7.11, 14.23)	309.79 (245.83, 389.98)	357.85 (294.06, 440.10)	355.15 (291.31, 439.60)	352.71 (290.52, 437.30)
American Samoa	13.41 (9.11, 18.51)	21.17 (14.83, 30.94)	22.34 (14.80, 31.79)	21.20 (14.95, 30.47)	393.64 (326.11, 472.41)	613.57 (495.63, 748.80)	646.88 (514.84, 792.67)	609.82 (477.72, 763.40)
Cook Islands	20.85 (13.46, 29.67)	29.68 (20.67, 41.95)	29.93 (20.62, 42.96)	30.27 (20.52, 44.52)	553.48 (425.89, 715.85)	581.03 (445.43, 744.02)	579.68 (436.04, 750.18)	579.63 (433.33, 755.30)
Fiji	15.33 (10.52, 21.75)	17.92 (12.44, 26.07)	18.05 (12.68, 26.14)	18.11 (12.30, 26.29)	507.55 (404.31, 633.53)	595.37 (454.36, 757.96)	595.60 (448.83, 766.57)	595.12 (440.78, 785.42)
Guam	10.69 (7.37, 15.03)	12.83 (8.68, 18.26)	11.69 (7.68, 16.15)	11.41 (7.40, 15.76)	243.54 (209.08, 283.08)	277.83 (232.59, 329.02)	251.26 (211.08, 298.02)	244.77 (204.08, 289.83)
Kiribati	9.40 (6.36, 13.22)	13.04 (8.36, 18.04)	13.10 (8.70, 18.09)	13.10 (8.75, 18.88)	393.51 (305.47, 500.61)	538.34 (407.15, 712.73)	539.46 (407.05, 718.60)	539.78 (406.17, 724.02)
Marshall Islands	8.96 (5.71, 13.27)	12.85 (6.94, 20.55)	12.90 (6.93, 20.76)	13.01 (6.92, 20.74)	331.78 (234.46, 469.37)	481.28 (287.97, 736.16)	481.08 (285.80, 745.20)	480.67 (287.91, 746.95)
Micronesia (Federated States of)	10.69 (6.72, 15.60)	15.14 (9.53, 22.13)	15.29 (9.60, 22.26)	15.33 (9.69, 22.49)	410.26 (279.11, 562.28)	527.31 (377.81, 700.04)	527.98 (375.75, 708.02)	527.04 (374.44, 705.27)
Nauru	12.40 (6.88, 20.42)	18.67 (10.23, 30.75)	18.85 (10.13, 31.01)	19.06 (10.18, 31.58)	452.17 (269.50, 694.44)	640.21 (383.74, 970.85)	640.61 (377.03, 978.63)	641.18 (378.14, 989.88)
Niue	14.31 (9.14, 20.29)	18.27 (11.91, 27.36)	19.75 (12.91, 29.22)	19.96 (13.12, 29.49)	433.77 (326.72, 569.31)	495.63 (359.61, 675.36)	533.79 (403.13, 706.39)	534.34 (402.43, 700.78)
Northern Mariana Islands	14.01 (9.21, 20.21)	17.70 (12.19, 25.78)	16.84 (11.35, 23.73)	17.96 (12.21, 25.14)	337.87 (250.53, 446.03)	399.32 (319.10, 509.73)	373.67 (291.56, 468.83)	404.21 (328.94, 467.72)
Palau	20.65 (13.66, 29.05)	21.57 (15.15, 31.55)	21.38 (14.98, 30.97)	21.39 (14.72, 30.67)	615.42 (468.80, 806.22)	572.19 (446.24, 735.95)	566.11 (438.42, 731.15)	564.63 (434.13, 727.06)
Papua New Guinea	7.05 (4.37, 10.49)	7.96 (5.22, 12.08)	7.98 (5.31, 12.21)	7.92 (5.17, 12.33)	264.24 (183.27, 371.44)	309.21 (226.35, 416.66)	307.50 (223.09, 417.65)	305.07 (220.19, 416.08)
Samoa	8.43 (5.64, 12.03)	11.00 (6.91, 16.32)	11.07 (6.90, 16.38)	11.15 (6.76, 16.75)	265.08 (203.12, 342.73)	325.40 (235.87, 434.07)	325.27 (235.07, 434.69)	324.98 (235.73, 433.84)
Solomon Islands	5.45 (3.21, 8.54)	9.21 (5.87, 13.91)	9.33 (5.80, 14.24)	9.27 (5.88, 13.84)	203.54 (125.32, 292.01)	354.22 (248.29, 481.25)	357.76 (250.46, 493.85)	352.38 (247.97, 478.49)
Tokelau	12.92 (7.78, 19.57)	16.81 (10.76, 24.94)	18.14 (11.85, 26.26)	18.32 (12.06, 26.34)	446.22 (288.34, 646.99)	480.60 (343.20, 653.95)	515.35 (389.68, 691.39)	514.13 (383.98, 685.29)
Tonga	17.25 (11.53, 24.37)	20.43 (13.48, 29.66)	20.70 (13.56, 29.67)	20.87 (13.93, 30.53)	539.22 (426.61, 683.87)	584.94 (435.21, 778.13)	586.38 (432.61, 778.32)	587.93 (428.99, 784.09)
Tuvalu	10.52 (6.25, 15.74)	13.57 (8.61, 19.70)	13.64 (8.77, 19.98)	13.64 (8.66, 19.87)	400.52 (263.55, 580.01)	451.42 (326.66, 616.88)	448.28 (319.95, 616.06)	444.77 (318.58, 615.28)
Vanuatu	5.88 (3.67, 9.05)	8.48 (5.69, 12.10)	8.66 (5.83, 12.39)	8.74 (5.94, 12.51)	207.33 (142.41, 295.90)	323.30 (241.64, 411.24)	326.74 (242.58, 416.77)	329.65 (240.57, 429.16)
Southeast Asia	6.70 (4.56, 9.24)	12.08 (8.14, 16.60)	12.09 (8.35, 16.83)	12.19 (8.38, 16.58)	234.11 (194.66, 283.64)	304.36 (258.86, 364.32)	301.79 (252.29, 364.81)	302.40 (251.48, 367.74)
Cambodia	5.91 (3.31, 9.49)	11.36 (7.57, 16.93)	11.52 (7.58, 17.24)	11.61 (7.62, 16.82)	244.55 (155.14, 370.45)	354.05 (259.97, 465.50)	354.65 (256.77, 469.81)	354.43 (252.33, 474.48)
Indonesia	6.32 (3.79, 9.86)	10.79 (6.12, 16.19)	10.89 (6.58, 16.96)	10.85 (6.68, 16.78)	233.32 (159.67, 328.27)	311.96 (210.21, 455.62)	311.36 (210.45, 454.72)	309.41 (209.24, 440.83)
Lao People’s Democratic Republic	5.35 (2.89, 8.84)	8.67 (5.63, 12.80)	8.76 (5.52, 13.02)	8.78 (5.57, 13.19)	237.16 (139.45, 383.91)	298.71 (215.70, 403.78)	298.00 (212.90, 408.00)	296.97 (210.53, 409.69)
Malaysia	10.22 (7.03, 14.32)	19.38 (13.45, 25.89)	18.39 (12.41, 24.89)	19.66 (13.36, 26.40)	331.19 (278.59, 388.19)	416.47 (352.16, 488.22)	391.23 (331.52, 461.98)	417.87 (353.51, 491.60)
Maldives	3.84 (1.86, 6.80)	6.22 (4.35, 8.94)	5.90 (4.05, 8.58)	5.98 (4.03, 8.90)	133.22 (70.49, 227.35)	115.35 (91.03, 141.87)	107.73 (84.41, 134.14)	108.47 (82.69, 136.81)
Mauritius	7.16 (5.07, 9.83)	18.33 (12.83, 24.71)	17.19 (12.21, 23.20)	18.25 (13.22, 24.72)	194.30 (181.03, 210.31)	389.70 (360.42, 419.44)	360.94 (332.32, 389.52)	381.63 (340.47, 410.01)
Myanmar	7.40 (4.26, 11.22)	10.81 (7.34, 15.76)	10.93 (7.25, 16.15)	11.00 (7.26, 16.71)	321.21 (217.21, 461.07)	335.67 (258.48, 442.17)	335.98 (257.56, 443.52)	335.93 (255.08, 446.40)
Philippines	8.71 (6.29, 11.63)	13.43 (9.46, 17.82)	13.45 (9.42, 17.89)	13.63 (9.15, 19.81)	305.31 (271.14, 337.79)	406.43 (362.15, 453.65)	401.57 (360.40, 445.95)	403.58 (315.99, 505.45)
Seychelles	8.97 (6.08, 12.43)	17.07 (12.01, 23.43)	16.16 (11.08, 22.22)	15.38 (10.84, 20.95)	269.51 (230.59, 312.07)	384.02 (331.08, 445.65)	358.07 (302.23, 418.70)	338.21 (287.03, 394.09)
Sri Lanka	5.66 (3.88, 7.95)	11.30 (7.35, 16.97)	10.65 (6.62, 16.51)	11.07 (6.74, 17.29)	168.40 (139.89, 200.45)	209.00 (152.17, 281.22)	192.73 (123.68, 263.98)	199.28 (130.24, 271.41)
Thailand	6.87 (4.65, 9.56)	16.40 (11.26, 22.95)	16.54 (11.20, 23.47)	16.60 (11.02, 24.08)	194.48 (158.13, 229.94)	284.95 (238.85, 337.85)	284.90 (222.63, 357.82)	283.61 (215.20, 361.70)
Timor-Leste	3.72 (2.14, 5.84)	6.42 (3.76, 9.89)	6.47 (3.89, 9.80)	6.50 (3.88, 10.10)	148.08 (93.82, 224.02)	213.23 (149.64, 293.85)	213.43 (148.75, 294.42)	214.24 (146.10, 291.92)
Viet Nam	5.05 (3.47, 7.35)	9.54 (6.37, 13.76)	9.70 (6.39, 13.83)	9.75 (6.29, 14.18)	157.06 (120.78, 205.96)	192.43 (147.43, 249.74)	192.57 (147.80, 251.82)	191.02 (143.97, 253.51)
Central Sub-Saharan Africa	5.49 (3.53, 8.06)	8.25 (5.57, 11.65)	8.45 (5.73, 12.13)	8.60 (5.74, 12.01)	235.79 (169.21, 313.00)	300.64 (230.90, 389.69)	305.87 (233.34, 398.42)	309.47 (232.95, 401.55)
Angola	4.83 (3.10, 7.02)	8.47 (5.47, 12.45)	8.72 (5.54, 12.96)	8.87 (5.60, 12.75)	210.11 (150.70, 290.33)	305.21 (219.86, 400.64)	310.78 (219.13, 413.27)	314.59 (216.72, 419.78)
Central African Republic	5.86 (3.73, 8.56)	6.87 (4.12, 9.88)	6.95 (4.24, 10.26)	7.01 (4.34, 10.17)	268.03 (193.27, 358.56)	300.21 (205.56, 412.25)	302.49 (209.40, 417.53)	304.62 (211.39, 418.14)
Congo	8.87 (4.66, 14.57)	13.33 (7.28, 22.01)	13.48 (7.32, 22.52)	13.59 (7.53, 22.71)	377.14 (224.62, 600.39)	456.77 (273.09, 704.70)	459.36 (276.78, 718.58)	459.27 (278.73, 725.25)
Democratic Republic of the Congo	5.28 (3.38, 7.71)	7.64 (5.03, 10.93)	7.82 (5.27, 11.60)	7.98 (5.20, 11.56)	226.19 (158.60, 307.51)	282.36 (208.03, 374.06)	287.89 (214.27, 388.59)	291.88 (216.62, 398.51)
Equatorial Guinea	5.74 (3.44, 8.78)	14.09 (7.65, 23.34)	14.44 (7.81, 23.94)	14.64 (8.07, 24.10)	252.98 (169.19, 363.95)	408.48 (230.50, 631.59)	413.82 (238.00, 649.94)	416.25 (243.77, 649.44)
Gabon	8.66 (5.43, 12.80)	13.54 (8.36, 20.53)	13.82 (8.62, 20.67)	13.94 (8.61, 20.70)	342.78 (244.62, 458.71)	414.71 (278.54, 568.93)	418.04 (279.25, 575.09)	417.96 (287.04, 577.29)
Eastern Sub-Saharan Africa	6.32 (4.42, 8.58)	9.26 (6.75, 12.40)	9.50 (6.75, 12.88)	9.64 (6.89, 12.74)	268.18 (224.54, 324.21)	327.38 (281.08, 384.35)	332.06 (281.73, 388.06)	335.78 (287.20, 390.50)
Burundi	6.71 (4.25, 9.99)	6.18 (4.13, 8.96)	6.19 (4.08, 9.01)	6.27 (4.21, 9.16)	294.14 (208.21, 415.32)	234.77 (174.76, 305.94)	235.73 (175.70, 311.03)	237.96 (174.56, 324.35)
Comoros	6.95 (4.51, 10.29)	10.47 (6.90, 15.48)	10.81 (6.91, 15.86)	10.94 (7.06, 15.73)	285.06 (205.77, 384.94)	367.66 (276.42, 479.07)	377.49 (285.19, 503.30)	379.64 (282.65, 501.22)
Djibouti	7.10 (4.80, 10.40)	9.66 (5.94, 14.90)	9.81 (6.12, 14.87)	10.03 (6.27, 15.36)	282.98 (209.71, 374.37)	329.43 (221.17, 482.05)	330.82 (224.56, 486.91)	335.57 (229.63, 487.60)
Eritrea	7.39 (4.72, 10.86)	10.63 (6.71, 15.49)	10.79 (6.60, 15.37)	10.89 (6.79, 15.30)	322.38 (238.19, 433.41)	411.44 (293.71, 560.84)	414.37 (294.05, 567.32)	417.10 (294.05, 570.77)
Ethiopia	6.47 (3.86, 10.39)	8.11 (5.59, 11.04)	8.36 (5.87, 11.52)	8.57 (5.81, 11.82)	297.65 (197.86, 439.62)	281.50 (229.51, 345.08)	286.99 (234.22, 357.44)	292.41 (238.68, 360.65)
Kenya	5.33 (3.44, 7.91)	9.85 (6.55, 14.44)	10.07 (6.51, 14.80)	10.24 (6.56, 15.30)	195.46 (139.33, 266.84)	322.09 (234.74, 441.74)	325.65 (234.22, 447.81)	327.97 (232.35, 447.09)
Madagascar	6.38 (4.31, 8.94)	7.77 (4.98, 11.18)	8.00 (5.24, 11.74)	8.12 (5.25, 12.25)	269.04 (213.01, 336.04)	285.86 (210.82, 380.76)	291.13 (213.11, 391.19)	295.79 (212.97, 396.54)
Malawi	5.27 (3.62, 7.35)	8.81 (5.82, 12.81)	9.00 (6.04, 13.09)	9.12 (6.08, 13.41)	218.89 (171.78, 270.38)	318.42 (232.19, 406.62)	321.68 (235.68, 411.46)	324.73 (235.51, 430.50)
Mozambique	5.47 (3.90, 7.54)	8.92 (5.64, 12.94)	9.02 (5.53, 13.38)	9.09 (5.70, 13.50)	230.05 (190.48, 282.46)	345.44 (248.73, 460.86)	346.16 (243.93, 452.77)	347.19 (245.89, 459.84)
Rwanda	8.79 (5.38, 13.63)	10.59 (7.01, 15.75)	10.91 (7.15, 16.17)	11.03 (7.02, 16.34)	405.59 (285.18, 572.18)	371.99 (262.33, 502.01)	378.21 (268.22, 519.84)	381.20 (266.75, 525.79)
Somalia	5.26 (3.30, 7.68)	5.94 (3.66, 8.90)	5.89 (3.79, 8.96)	5.90 (3.76, 8.93)	230.73 (157.81, 315.17)	245.04 (167.01, 339.90)	244.87 (167.15, 336.17)	244.50 (162.66, 335.65)
South Sudan	4.88 (3.27, 7.06)	6.64 (4.17, 9.94)	6.76 (4.27, 10.21)	6.92 (4.36, 10.36)	202.12 (142.20, 284.66)	243.18 (169.22, 343.08)	248.40 (173.92, 353.46)	252.84 (177.75, 361.34)
Uganda	7.19 (4.66, 10.35)	12.40 (8.33, 18.10)	12.82 (8.68, 18.82)	13.03 (8.65, 19.18)	286.09 (208.33, 381.61)	433.81 (326.54, 574.18)	443.07 (326.31, 591.95)	448.65 (328.45, 596.41)
United Republic of Tanzania	6.93 (4.68, 9.62)	9.73 (6.47, 13.66)	10.00 (6.74, 14.39)	10.12 (6.69, 14.55)	278.03 (223.58, 336.77)	336.22 (254.18, 442.13)	340.92 (254.77, 449.68)	345.05 (254.41, 453.77)
Zambia	6.56 (4.14, 9.65)	14.11 (7.96, 24.01)	14.40 (8.13, 24.05)	14.38 (8.11, 23.60)	280.95 (203.57, 385.89)	512.90 (303.22, 805.46)	515.53 (306.92, 808.35)	512.85 (303.94, 786.57)
Southern Sub-Saharan Africa	8.49 (6.02, 11.71)	13.95 (10.25, 18.29)	14.30 (10.60, 18.81)	14.23 (10.48, 18.55)	286.48 (240.24, 333.58)	407.21 (370.89, 449.84)	415.37 (378.12, 457.46)	412.57 (370.37, 457.27)
Botswana	7.31 (4.63, 10.92)	10.71 (7.12, 16.44)	10.75 (7.20, 16.49)	10.68 (7.09, 16.40)	275.42 (188.22, 384.27)	338.42 (241.36, 468.97)	336.44 (242.22, 462.99)	333.91 (237.72, 475.98)
Eswatini	7.42 (4.97, 10.37)	13.09 (7.27, 21.32)	13.04 (7.26, 20.80)	13.04 (7.36, 21.11)	272.60 (206.70, 348.97)	448.97 (259.35, 694.57)	445.15 (258.07, 686.70)	442.52 (257.35, 684.06)
Lesotho	6.06 (3.87, 8.88)	11.94 (7.17, 18.75)	11.92 (7.02, 18.20)	11.77 (6.74, 18.22)	223.40 (155.08, 312.03)	453.02 (284.85, 672.27)	451.16 (285.15, 670.22)	444.13 (282.04, 650.98)
Namibia	7.97 (5.46, 11.11)	17.75 (10.87, 26.70)	17.84 (10.99, 27.58)	17.81 (10.88, 26.72)	302.72 (251.52, 372.08)	538.21 (352.69, 737.06)	537.80 (348.83, 747.36)	535.85 (349.41, 754.75)
South Africa	8.93 (6.16, 12.54)	14.00 (10.32, 18.46)	14.43 (10.66, 19.29)	14.36 (10.58, 19.18)	297.01 (240.57, 355.18)	386.54 (345.36, 429.79)	397.00 (356.84, 439.56)	393.54 (353.97, 437.10)
Zimbabwe	7.09 (4.74, 10.02)	13.84 (9.17, 19.80)	13.80 (9.16, 19.55)	13.78 (9.09, 19.92)	246.15 (190.29, 317.58)	506.00 (376.59, 670.51)	504.30 (369.07, 667.98)	504.68 (363.77, 678.11)
Western Sub-Saharan Africa	5.90 (4.10, 8.17)	10.02 (6.77, 14.43)	10.22 (6.80, 14.44)	10.45 (7.28, 14.59)	241.41 (199.08, 285.82)	347.83 (272.80, 447.47)	351.19 (259.20, 446.46)	356.82 (276.17, 457.30)
Benin	4.42 (3.07, 6.06)	5.80 (3.78, 8.36)	6.00 (3.96, 8.84)	6.08 (4.02, 8.98)	181.82 (146.09, 217.53)	204.06 (148.43, 268.58)	208.58 (150.26, 275.56)	209.64 (152.51, 278.02)
Burkina Faso	7.54 (5.05, 10.50)	9.44 (6.09, 13.09)	9.41 (6.14, 13.45)	9.48 (6.17, 13.73)	315.16 (239.86, 398.42)	344.55 (242.99, 448.91)	342.07 (242.77, 448.73)	342.31 (244.77, 453.42)
Cabo Verde	7.75 (5.25, 10.89)	9.30 (6.62, 13.12)	9.46 (6.46, 13.49)	9.46 (6.54, 13.79)	260.70 (206.23, 322.18)	221.75 (174.53, 278.77)	222.51 (172.81, 284.41)	220.81 (169.48, 284.81)
Cameroon	5.82 (3.98, 8.20)	8.05 (5.20, 12.19)	8.17 (5.07, 12.42)	8.31 (5.20, 12.71)	234.32 (187.36, 289.31)	278.24 (198.41, 383.57)	279.58 (198.63, 385.44)	281.68 (201.25, 388.49)
Chad	3.63 (2.39, 5.25)	4.60 (2.97, 6.72)	4.68 (2.98, 6.87)	4.75 (3.03, 6.95)	150.06 (108.23, 196.59)	181.06 (131.16, 240.50)	183.06 (132.19, 243.92)	184.74 (131.95, 247.10)
Côte d’Ivoire	6.00 (4.15, 8.70)	9.37 (6.19, 13.88)	9.56 (6.32, 14.42)	9.72 (6.37, 14.82)	243.18 (190.72, 311.47)	317.12 (233.24, 426.60)	319.47 (232.01, 429.23)	320.78 (232.42, 432.76)
Gambia	2.57 (1.71, 3.73)	4.65 (3.00, 6.68)	4.67 (3.11, 6.70)	4.73 (3.04, 6.92)	91.54 (67.69, 121.24)	154.86 (113.59, 203.45)	153.81 (112.55, 203.20)	155.13 (113.19, 207.72)
Ghana	7.12 (4.78, 10.20)	10.22 (6.68, 14.63)	10.41 (6.83, 15.16)	10.57 (7.01, 15.19)	280.12 (215.59, 353.82)	328.11 (244.44, 435.88)	329.91 (247.52, 440.71)	330.68 (244.20, 437.73)
Guinea	4.64 (3.12, 6.44)	6.72 (4.32, 9.83)	6.85 (4.45, 10.02)	6.97 (4.47, 10.21)	197.17 (148.78, 247.02)	257.85 (182.86, 350.05)	260.64 (184.53, 357.55)	263.17 (186.05, 366.86)
Guinea-Bissau	5.62 (3.47, 8.36)	8.45 (5.35, 13.03)	8.50 (5.36, 13.07)	8.59 (5.42, 12.95)	256.02 (176.34, 375.42)	336.69 (239.25, 455.65)	337.83 (238.63, 458.50)	338.52 (236.22, 463.19)
Liberia	4.00 (2.72, 5.54)	6.73 (4.36, 10.31)	6.90 (4.48, 10.51)	7.04 (4.49, 11.12)	167.92 (131.06, 209.65)	227.82 (161.26, 319.78)	231.93 (161.08, 328.79)	235.08 (163.23, 333.64)
Mali	5.41 (3.74, 7.44)	7.02 (4.56, 10.54)	7.09 (4.62, 10.57)	7.17 (4.65, 10.70)	229.61 (188.02, 276.45)	255.82 (184.97, 345.02)	256.30 (183.53, 350.61)	258.13 (185.97, 354.31)
Mauritania	5.66 (3.54, 8.26)	8.82 (5.75, 13.01)	9.06 (6.03, 13.50)	9.39 (6.27, 13.82)	224.55 (161.91, 307.20)	258.66 (195.24, 337.16)	262.86 (192.27, 346.30)	272.25 (205.77, 362.89)
Niger	3.10 (2.07, 4.47)	3.87 (2.36, 5.84)	3.92 (2.49, 6.06)	3.95 (2.41, 6.03)	129.49 (97.21, 174.06)	146.29 (102.63, 206.52)	147.28 (101.64, 207.28)	147.83 (101.00, 204.57)
Nigeria	6.35 (4.26, 9.36)	12.61 (7.90, 19.01)	12.90 (7.97, 19.78)	13.24 (8.44, 19.70)	261.17 (194.97, 343.39)	438.15 (304.37, 616.43)	443.42 (285.48, 636.45)	453.39 (309.70, 650.22)
Sao Tome and Principe	4.85 (3.32, 6.83)	9.04 (5.91, 12.85)	9.22 (6.24, 13.46)	9.31 (6.23, 13.64)	174.32 (142.03, 212.55)	261.95 (193.71, 340.79)	263.62 (197.20, 346.21)	263.43 (199.02, 347.78)
Senegal	4.57 (3.11, 6.40)	7.68 (5.07, 11.20)	7.79 (5.03, 11.38)	7.88 (5.26, 11.45)	179.86 (141.22, 224.72)	259.11 (193.77, 344.91)	260.49 (195.75, 347.05)	261.15 (196.97, 347.57)
Sierra Leone	3.82 (2.46, 5.52)	6.26 (4.13, 9.44)	6.33 (4.15, 9.39)	6.43 (4.14, 9.46)	152.60 (106.66, 204.60)	222.87 (159.05, 296.52)	224.30 (160.19, 296.65)	225.79 (160.80, 298.42)
Togo	5.45 (3.74, 7.73)	8.28 (5.44, 12.61)	8.46 (5.46, 12.84)	8.61 (5.56, 13.07)	215.30 (172.40, 271.61)	283.41 (202.82, 386.70)	285.83 (205.25, 386.34)	288.94 (206.47, 391.06)

#### Global and regional distribution of breast cancer-related YLDs

We further analyzed the years lived with disability (YLDs) attributable to breast cancer across global, SDI, and regional levels (Table 1; Figures S1–S2). Globally, the age-standardized YLDs rate increased slightly from 15.28 (11.00, 20.36) in 1990 to 17.07 (12.30, 23.27) per 100,000 in 2021. By SDI strata, High and High-middle SDI regions maintained low but rising YLDs rates over the 31-year period, whereas Low and Low-middle SDI regions exhibited higher baseline YLDs rates with steady increases. Figure S1 illustrates the 2021 YLDs rates across three age groups: 15–49, 50–74, and 75 years and older. YLDs rates rose markedly with advancing age, with the highest values observed in the 75 + age group, consistent with the age patterns of DALYs. Figure S2 presents long-term trends in age-standardized YLDs rates from 1990 to 2021 at the global level and across 21 GBD regions. Most regions showed continuous growth in YLDs, especially in Western Sub-Saharan Africa, South Asia, and Southeast Asia. In contrast, high-income regions including Western Europe, High-income North America, and Australasia maintained relatively high YLDs rates but slower growth or mild declines in recent years. Detailed national and regional estimates of YLDs and corresponding 95% UIs are presented in Table 1.

### Analysis of trends in age-standardized DALYs for breast cancer: an international comparison from 1990 to 2021

**Fig. 1:**
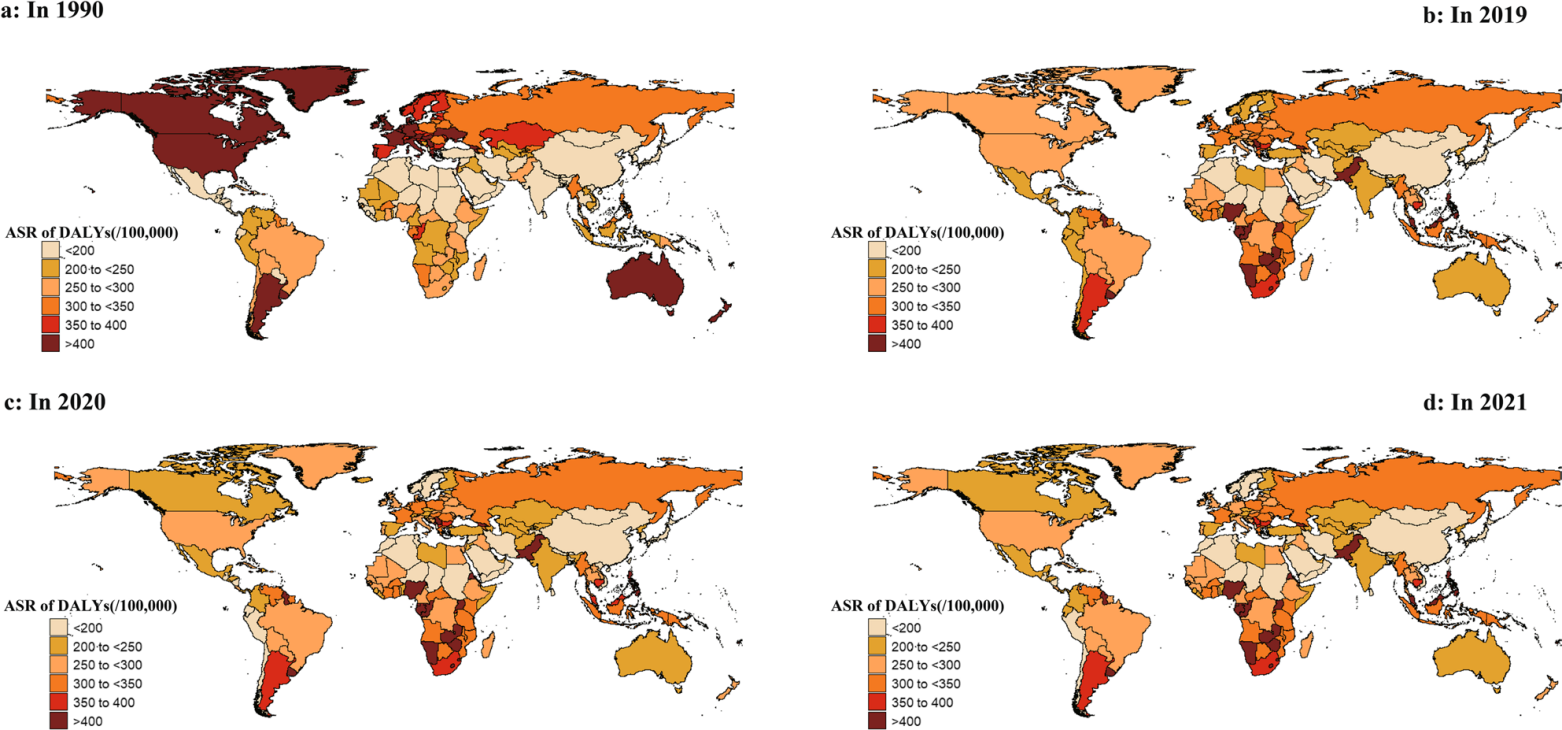
The DALYs trend of age standardization of breast cancer from 1990 to 2021

During this period, low-income and middle-income social development index (SDI) countries showed a growth trend, while high-income and high-middle SDI countries showed a downward trend (Fig. 1). Specifically, there are 26 countries with growth rates exceeding 50%, with Turkey having the highest growth rate, with its DALYs increasing from 96.16 per 100,000 to 214.05 per 100,000. On the other hand, there are three countries with a decline of over 50%, namely Denmark, Luxembourg, and United Kingdom. During the epidemic period of novel coronavirus from 2019 to 2021, there are 63 countries (accounting for 31%) with DALYs rising for three consecutive years, most of which are located in Africa. The country with the largest increase is Niue, with a cumulative growth rate of 38%. 75 countries (37%) have experienced a continuous decline for three years, with the majority located in Europe and the United States. Among them, 5 countries have experienced a decline of over 50%, namely United States Virgin Islands, San Marino, United Arab Emirates, Andorra, and Antigua and Barbuda. In addition, 14 countries (7%) showed a trend of first increasing and then decreasing, with the majority located in Oceania and the United States; However, 51 countries (accounting for 25%) showed a trend of first decreasing and then increasing, with the majority located in Europe and the United States.

### Sex and geographical differences in the global epidemiology of breast cancer

**Fig. 2:**
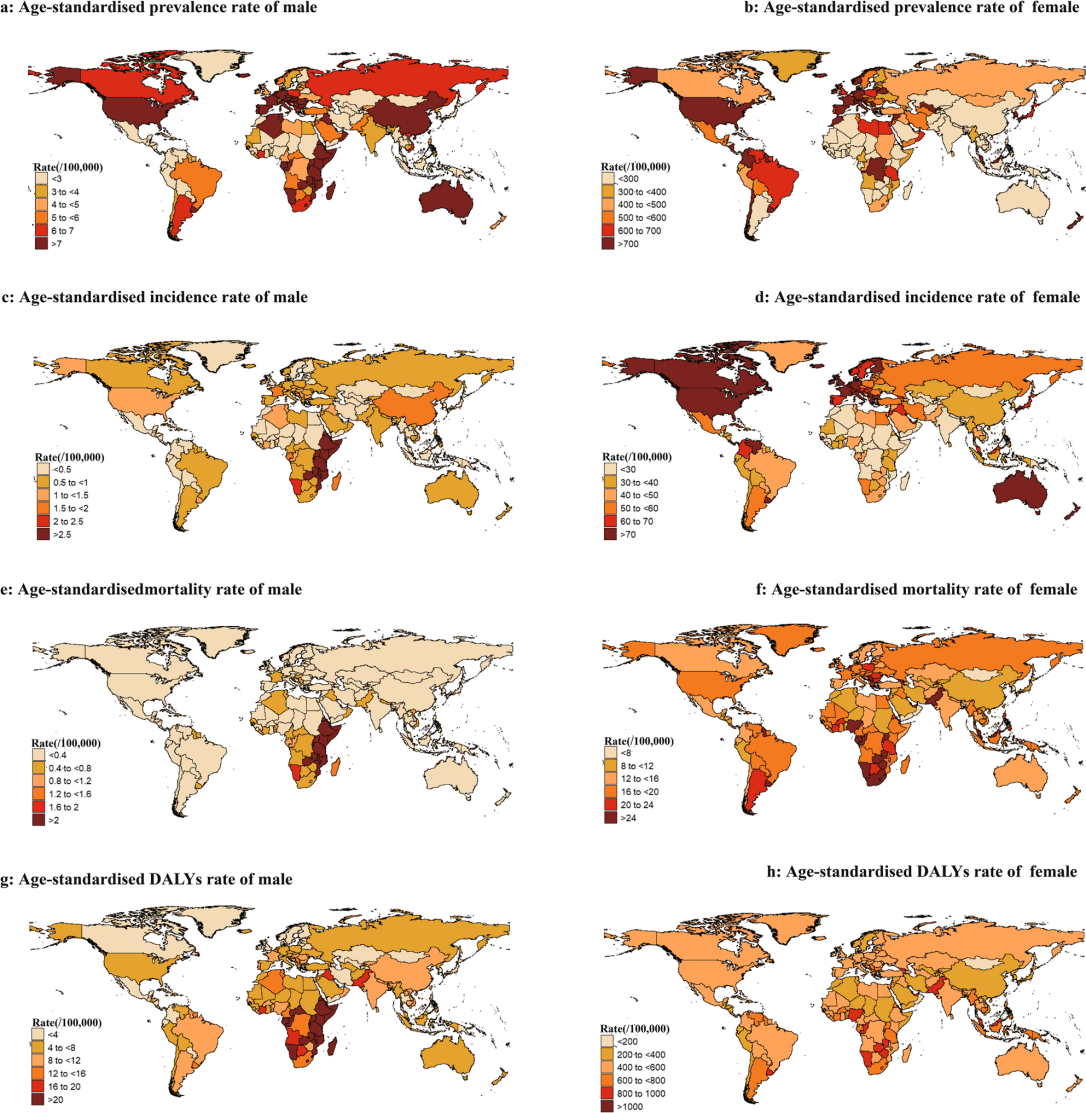
The gender differences in the prevalence, incidence rate, mortality and age standardized rate of DALYs of male and female breast cancer in 2021

Figure 2 shows the sex difference in the age-standardized rates of prevalence, incidence, mortality, and DALYs due to breast cancer in 2021. As for prevalence in males, 8 out of the top 10 countries are in Eastern Sub-Saharan Africa; for females, 5 out of the top 10 countries are in Western Europe. Regarding incidence in males, 9 out of the top 10 countries are in Eastern Sub-Saharan Africa; for females, 4 out of the top 10 countries are in North Africa and the Middle East, and three are in the Caribbean region. For mortality in males, 8 out of the top 10 countries are in Eastern Sub-Saharan Africa; for females, 5 out of the top 10 countries are in Oceania. Concerning DALYs in males, all top 10 countries are in Eastern Sub-Saharan Africa; for females, 8 out of the top 10 countries are in Oceania. Overall, the data indicate that the trends for males and females are not consistent and exhibit significant differences.

Male breast cancer has a very low global incidence, and the burden estimates in countries with small populations (such as Nauru, Palau, Niue, etc.) have wide 95% UIs, indicating high uncertainty of the estimates, which should be interpreted with caution. The core conclusions of male breast cancer burden are based on regional-level aggregated results, which have a larger sample size, narrower 95% UIs, and lower uncertainty.

### Interesting patterns revealed by global age differences in breast cancer DALYs

**Fig. 3:**
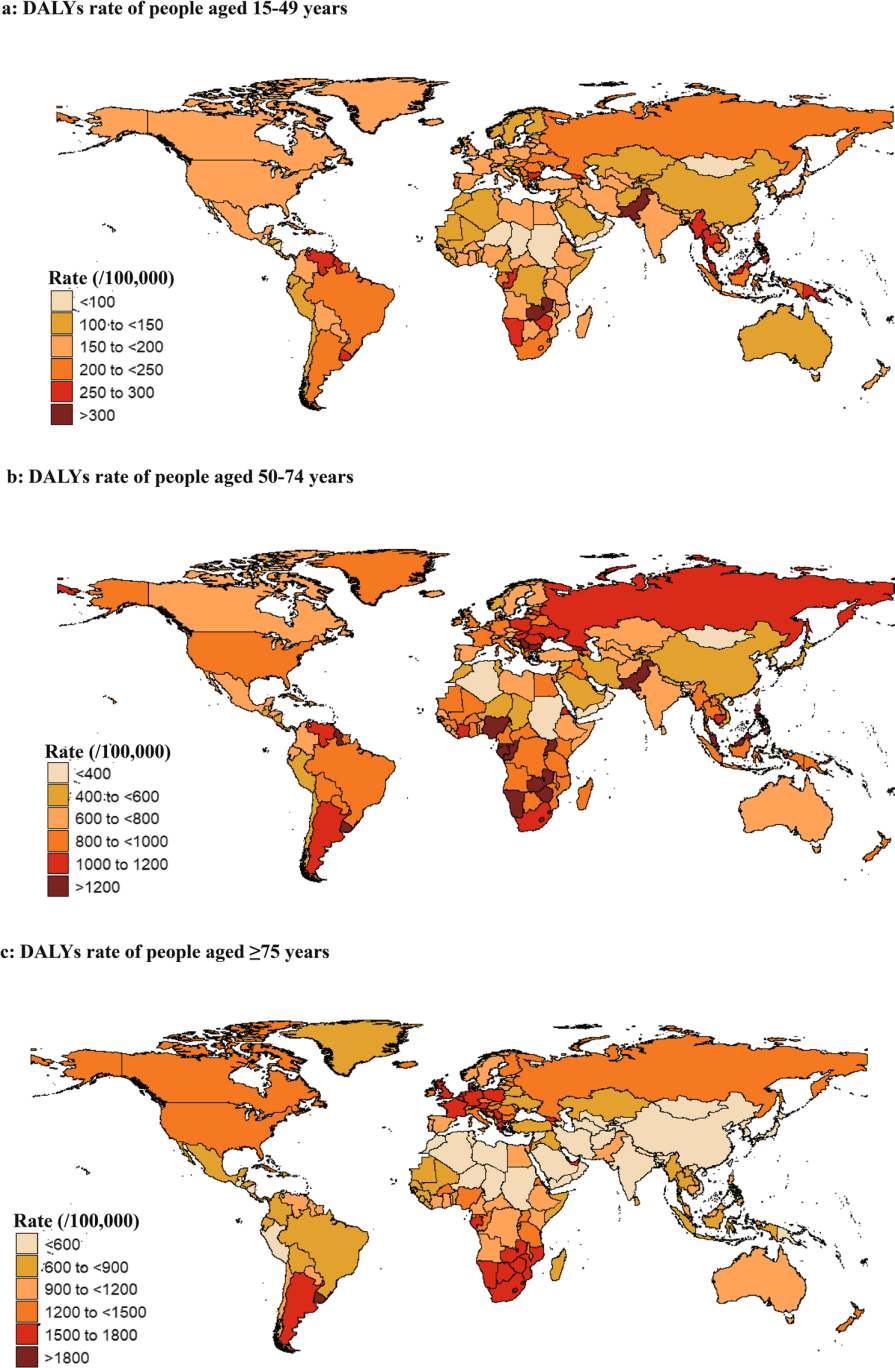
Global disparities in breast cancer DALYs across age groups

To further analyze the differences in DALYs among breast cancer patients of different age groups, we divided all breast cancer patients into high, middle, and low age groups (Fig. 3). The high age group includes those aged 75 and above, the middle age group includes those aged 50 to 74, and the low age group includes those aged 15 to 49. In terms of SDI, breast cancer patients in the 15–49 years age group have the highest DALYs in low-middle SDI countries, approximately 180.86 per 100,000 (95% UI: 161.08, 201.32). For the 50–74 years age group, the highest DALYs are found in low SDI countries, about 754.47 per 100,000 (95% UI: 662.29, 853.46). In the ≥ 75 years age group, the highest DALYs are observed in high SDI countries, approximately 1166.46 per 100,000 (95% UI: 952.49, 1296.24).

At the national level, for the low age group, the highest DALYs are in the Bahamas, approximately 504.22 per 100,000 (95% UI: 383.26, 652.45), and the lowest in Oman, approximately 24.84 per 100,000 (95% UI: 17.66, 33.84). For the middle age group, the highest DALYs are in Nauru, approximately 1041.61 per 100,000 (95% UI: 662.29, 2744.46), and the lowest in Oman, approximately 204.83 per 100,000 (95% UI: 147.19, 272.24). For the high age group, the highest DALYs are in Palau, approximately 2906.13 per 100,000 (95% UI: 2301.60, 3658.84), and the lowest in Bangladesh, approximately 55.22 per 100,000 (95% UI: 41.28, 73.25).

### Social development indicators and breast cancer indicators: complex transnational and regional relationships

**Fig. 4:**
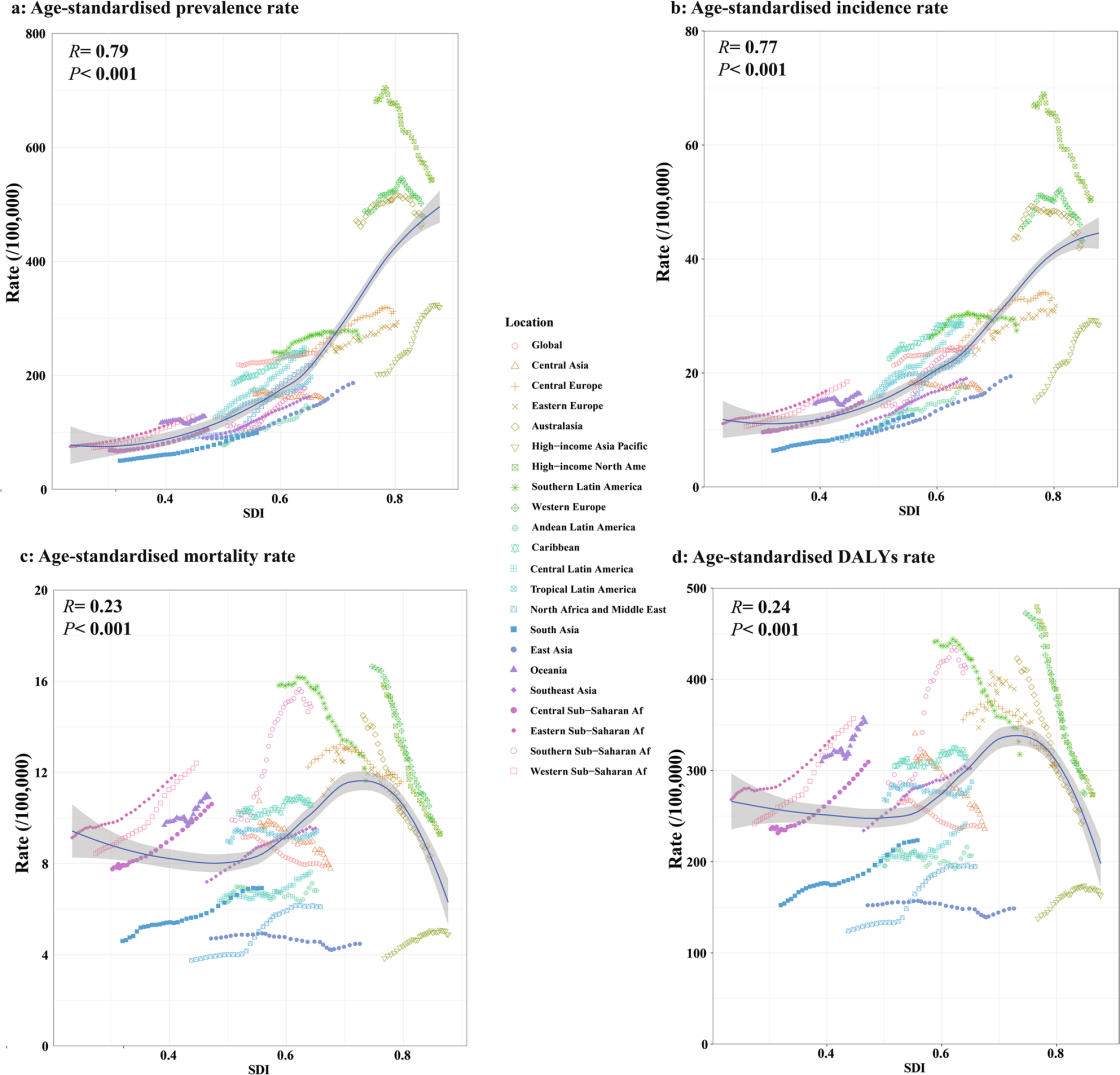
Associations between SDI Levels and Breast Cancer Prevalence, Incidence, Mortality, and DALYs Across Different Countries and Regions

We evaluated the association between SDI levels and the DALYS of breast cancer in different countries and regions. Overall, the highest prevalence and incidence rates were observed in the very high SDI group, and the lowest occurred in the very low SDI group (Fig. 4a and b). The mortality and DAYLS rate initially increased with the increase in SDI but reached a peak and started to decrease in countries with a very high HDI (> 0.7) (Fig. 4c and d). Certainly, there are some exceptional countries and regions. In East Asia, where the SDI ranges between 0.45 and 0.75, the prevalence and incidence rates exhibit consistency with the overall trend. However, the mortality and DALYs remain showed a stable trend and do not vary with changes in SDI. For Central Asia and Southern Sub-Saharan Africa, with SDI falling in the Low-Middle and Middle categories, the prevalence and incidence rates maintain a steady state. Meanwhile, the mortality and DALYs decrease as SDI increases. In High-income Asia Pacific, mortality and DALYs exhibit an opposite trend compared to the overall pattern, increasing with rising SDI.

### Analysis of DALYs trends: global and regional differences

**Fig. 5:**
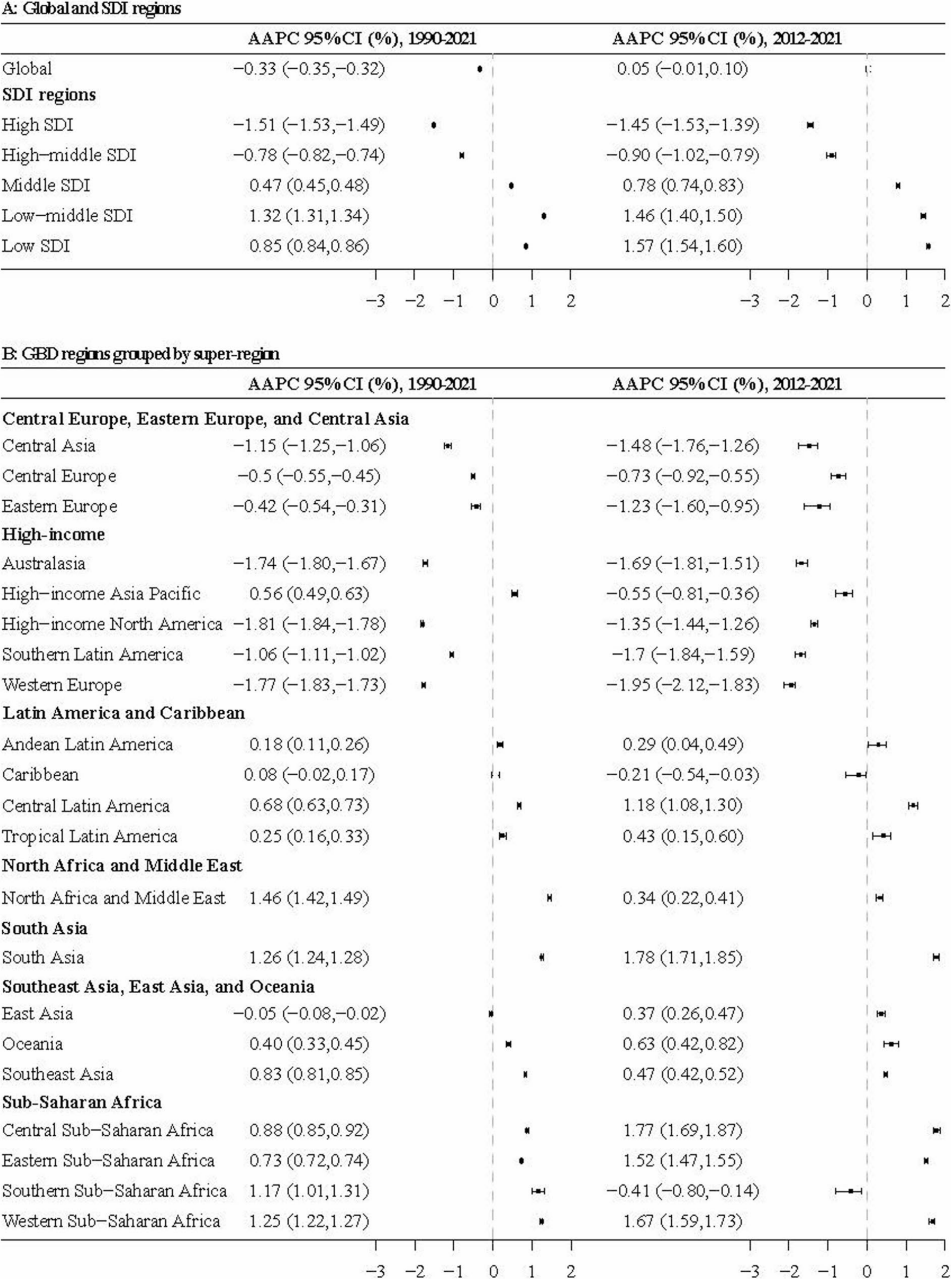
Annual average percentage changes (AAPCs) in disability-adjusted life years (DALYs) across global, SDI-stratified, and GBD super-regional levels, 1990–2021 and 2012–2021. **A** AAPCs and corresponding 95% confidence intervals (CIs) of DALYs at the global level and across five SDI (Socio-demographic Index) regions for the periods 1990–2021 and 2012–2021. **B** AAPCs and 95% CIs of DALYs across 21 GBD sub-regions, grouped by seven GBD super-regions, for the two study periods. Negative AAPC values indicate a decreasing trend in DALYs, while positive values indicate an increasing trend. Trends were considered statistically significant when the 95% CI did not include zero

To further analyze the changes in DALYs over the past decade, we compared them to the 30-year DALYs trends (Fig. 5a and b). For global and SDI regional trends, DALYs demonstrated a statistically significant slight decrease over the 30-year period from 1990 to 2021 (AAPC = − 0.33%, 95% CI:−0.35% to − 0.32%). In contrast, the recent decade (2012–2021) showed a non-significant near-stable trend, with an AAPC of 0.05% (95% CI:−0.01% to 0.10%) where the 95% CI crossed the null value. Substantial disparities in DALYs trends were evident across SDI strata. High and high-middle SDI regions exhibited consistent statistically significant decreasing trends in both time periods. High SDI regions had AAPCs of − 1.51% (95% CI: −1.53% to − 1.49%) in 1990–2021 and − 1.45% (95% CI: −1.53% to − 1.39%) in 2012–2021. High-middle SDI regions showed a slightly accelerated decline in the recent decade, with AAPCs of − 0.78% (95% CI: −0.82% to − 0.74%) in 1990–2021 and − 0.90% (95% CI:−1.02% to − 0.79%) in 2012–2021. Conversely, middle, low-middle, and low SDI regions all experienced statistically significant increasing trends in both periods, with a pronounced acceleration in the 2012–2021 period. Middle SDI regions had AAPCs of 0.47% (95% CI: 0.45% to 0.48%) in 1990–2021 and 0.78% (95% CI: 0.74% to 0.83%) in 2012–2021. Low-middle SDI regions had AAPCs of 1.32% (95% CI: 1.31% to 1.34%) and 1.46% (95% CI: 1.40% to 1.50%) for the two periods, respectively. Low SDI regions showed the most dramatic acceleration, with the AAPC nearly doubling from 0.85% (95% CI: 0.84% to 0.86%) in 1990–2021 to 1.57% (95% CI: 1.54% to 1.60%) in 2012–2021(Figure 5A).

As for GBD Super-Regional Trends, results were showed in Fig. 5B. Among the 21 GBD sub-regions, three regions showed a reversal in DALYs trends between the two study periods. High-income Asia Pacific shifted from a statistically significant increase in 1990–2021 (AAPC = 0.56%, 95% CI: 0.49% to 0.63%) to a significant decrease in 2012–2021 (AAPC = − 0.55%, 95% CI:−0.81% to − 0.36%). Southern Sub-Saharan Africa also reversed from a significant increase (AAPC = 1.17%, 95% CI: 1.01% to 1.31%) to a significant decrease (AAPC = − 0.41%, 95% CI:−0.80% to − 0.14%). The Caribbean region exhibited a non-significant increasing trend in 1990–2021 (AAPC = 0.08%, 95% CI:−0.02% to 0.17%) but transitioned to a statistically significant decrease in 2012–2021 (AAPC = − 0.21%, 95% CI:−0.54% to − 0.03%). Notably, East Asia displayed the opposite reversal pattern. It had a statistically significant slight decrease in DALYs during 1990–2021 (AAPC = − 0.05%, 95% CI:−0.08% to − 0.02%) but shifted to a significant increase in the recent decade (AAPC = 0.37%, 95% CI: 0.26% to 0.47%). Consistent with the observed DALYs trends, the incidence, prevalence, and mortality rates in the three regions that shifted from increasing to decreasing DALYs also demonstrated downward trends during 2012–2021 compared to the 1990–2021 period.

## Discussion

This study used data from the GBD 2021 study to estimate the diversity of disability due to breast cancer in 204 countries and territories and to further assess breast cancer incidence, prevalence, mortality, and life years of disability. Our analysis shows that DALYs generally decline in developed countries with high economy. Considering that developed countries usually have better medical system, more advanced medical technology and higher health investment, these countries can detect diseases earlier and treat patients more effectively, thus reducing DALYs [26]. However, DALYs increase in developed countries with low economy. Considering that developed countries with low economy often face problems such as shortage of medical resources and backward medical facilities, diseases are often difficult to get timely and effective treatment, which leads to the increase of DALYs [27]. It is also worth noting that there are regional differences in the global change trend of DALYs during the epidemic period, with DALYs generally rising in African countries and a significant downward trend in Europe and the United States.

Our global analysis of sex and geographical differences in breast cancer shows that sub-Saharan Africa is the region with the highest incidence of male breast cancer and Western Europe is the region with the highest incidence of female breast cancer [28]. This difference is not only reflected in morbidity, but also in prevalence, mortality, and DALYs. The high incidence and mortality of male breast cancer in Eastern Sub-Saharan Africa may be due to the low access to cancer screening, early diagnosis and treatment due to the general inadequacy of medical resources in the region, resulting in many patients missing the optimal time for treatment [29]. The incidence of female breast cancer is higher in Western Europe, which may be related to factors such as European lifestyle, diet, and aging population. In addition, the largest causes of high mortality and DALYs in Oceania are also related to unequal distribution of medical resources, inadequate access to treatment, cultural differences, and socioeconomic factors.

This study revealed several key new insights that advance existing research: First, the age-SDI interaction characteristics of breast cancer disability burden: the peak burden of the 15–49 years age group is in low-middle SDI countries, the 50–74 years age group in low SDI countries, and the ≥ 75 years age group in high SDI countries, which provides a precise direction for different countries to formulate age-specific breast cancer screening and rehabilitation intervention strategies. Second, the “two-way differentiation” trend of global breast cancer DALYs during the COVID-19 pandemic: 63% of African countries experienced a continuous increase for 3 years, while 75% of high-income countries in Europe and the United States experienced a continuous decline, which provides data support for the differentiated allocation of global breast cancer prevention and control resources in the post-pandemic era. Third, the core driving differences of breast cancer burden between high SDI and low SDI countries: high SDI countries are dominated by disease-related disability burden of the elderly population, while low SDI countries are dominated by premature death-related DALYs of young and middle-aged populations, which provides a basis for the two types of countries to formulate different prevention and control priorities.

The high DALYs in low and middle age groups in low- and middle-income countries may be attributed to a series of reasons. For example, low- and middle-income countries often face the problem of shortage of medical resources, including the shortage of doctors, nurses, medical equipment and drugs; Factors such as poverty, low education levels and poor lifestyle habits in low- and middle-income countries may lead to higher exposure to health risks in lower age groups [30]. Part of the reason for the high DALYs in high-income countries is that high-income countries are often faced with the problem of population aging, that is, the proportion of elderly people is gradually increasing. Status of breast cancer screening in developing countries pointed out that due to limited medical resources and insufficient awareness of screening, the early diagnosis rate of breast cancer in developing countries is generally low, resulting in relatively high rates of disability and mortality. This highlights the importance of strengthening breast cancer screening and health education globally [31]. With the growth of age, elderly people are more likely to suffer from chronic diseases, such as cardiovascular diseases, diabetes, cancer, etc. These diseases usually require long-term medical care and rehabilitation. Therefore, DALYs will increase. In addition, higher income countries have more advanced medical technology and diagnostic capacity, which allows the diagnosis of some diseases that might otherwise go undetected, which may increase DALYs, but also provide patients with more treatment options and a better quality of life. Research by GBD 2019 Viewpoint Collaborators (2020) reveals multi-dimensional perspectives on global health challenges, providing a macro framework for understanding the global context of breast cancer disability. These insights underscore the critical role of international cooperation in addressing global health issues [32].

Global DALYs have generally remained stable over the past decade, but regions with different SDI levels show different trends, with high-income Asia Pacific, the Caribbean, and sub-Saharan Africa showing a declining trend in DALYs between 2012 and 2021, while East Asia shows the opposite trend. Research by GBD 2019 Mental Disorders Collaborators (2022) focuses directly on mental disorders, but its methodology provides valuable references for analyzing health issues such as breast cancer disability, such as the application of geospatial technology in health monitoring [33].

There are several advantages and limitations to consider when interpreting our results. The strengths of our analysis include that our study is based on a large amount of international data, covering multiple countries and regions with different levels of income and social development, making the results more generic and comparable. Most importantly, our study covers a longer time period (from 1990 to 2021), so trends in disease burden (such as DALYs for breast cancer) can be observed over time, providing a historical reference for policy formulation and intervention strategies. There are some limitations to our study. First, there may be differences in data quality and availability in different countries and regions, which may lead to data inconsistencies and bias, affecting the accuracy of research results. Second, studies may not accurately predict how the burden of breast cancer will change in the future, because many factors (such as technological advances, demographic changes, policy adjustments, etc.) can affect the burden of the disease. Kamel Boulos M and Wilson JP highlighted the potential of geospatial technologies for monitoring and mitigating climate change and its impacts on human health. Similarly, these technologies can be used to map the geographic distribution of breast cancer disability, providing spatial support for precision medicine [34]. Therefore, the predictive value of the findings may be limited. In addition, male breast cancer has a very low global incidence, and the burden estimates in countries with small populations have wide 95% UIs and high uncertainty, so the relevant results should be interpreted with caution, and the core conclusions should be based on regional-level aggregated data.

Taken together, this study finds that the global burden of breast cancer remains significant and poses a serious threat to women’s health across the globe. The global burden of disability due to breast cancer varies significantly across countries and regions. DALYs for breast cancer have increased in some countries during the COVID-19 pandemic, particularly in low - and middle-income countries where resources are less available. This finding highlights the importance of strengthening breast cancer prevention, early screening and treatment strategies globally, especially in resource-limited regions. At the same time, attention and research on male breast cancer should also be increased, especially in sub-Saharan Africa.

Based on our findings, we propose targeted actionable strategies for countries across different SDI strata to reduce breast cancer disability burden. For high and high-middle SDI countries: [1] Continuously optimize the breast cancer early screening system, focus on covering the elderly population (≥ 75 years old), and promote individualized screening strategies based on risk stratification; [2] Improve the whole-cycle rehabilitation management system for breast cancer survivors, reduce long-term treatment-related disability, and lower YLDs burden; [3] Strengthen the population-based prevention and control of breast cancer risk factors, including health education on controllable risk factors such as obesity, lack of exercise, and alcohol consumption. For middle SDI countries: [1] Gradually expand the population coverage of breast cancer screening, focus on middle-aged women aged 50–74, and improve the early diagnosis rate; [2] Improve the standardized system of breast cancer diagnosis and treatment, enhance the diagnosis and treatment capacity of primary medical institutions, and reduce disability and premature death caused by non-standard diagnosis and treatment; [3] Establish a monitoring system for breast cancer incidence and mortality, and dynamically grasp the changing trend of disease burden. For low-middle and low SDI countries: [1] Implement low-cost and easy-to-popularize breast cancer early diagnosis strategies, such as clinical breast examination (CBE), to replace high-cost mammography screening and improve primary accessibility; [2] Strengthen health education on core symptoms of breast cancer, improve women’s medical seeking awareness, and reduce the proportion of advanced cases; [3] Establish a security system for basic breast cancer diagnosis and treatment, include basic treatment drugs such as endocrine therapy into the national essential medicine list, and improve treatment accessibility.

Core difficulties faced by low-income countries in reducing breast cancer burden include: [1] Severe shortage of medical resources: Primary medical institutions lack basic equipment and professional medical staff for breast cancer screening and diagnosis. Most patients cannot obtain early diagnosis and standardized treatment, and are diagnosed at an advanced stage, which is the core cause of high DALYs [2]. Weak health security system: Most low-income countries lack universal medical insurance, and the cost of breast cancer diagnosis and treatment causes catastrophic medical expenditure for families, and a large number of patients give up treatment due to economic reasons [3]. Insufficient health literacy: The population has insufficient awareness of the early symptoms of breast cancer and the importance of screening, and serious disease stigma leads to delayed medical treatment [4]. Constraints of socio-economic development: Low-income countries face the dual disease burden of infectious and non-communicable diseases. Public health resources are preferentially allocated to the prevention and control of infectious diseases, and the investment in the prevention and control of non-communicable diseases such as breast cancer is seriously insufficient [5]. Superimposed impact of the pandemic: The COVID-19 pandemic further crowded the already limited medical resources, interrupted routine breast cancer screening and treatment, leading to a continuous rise in breast cancer burden in low-income countries, and further aggravated the difficulty of prevention and control.

## Conclusion

The Global Burden of Disease Study 2021 provides a valuable snapshot of the diverse impact of breast cancer on disability across 204 countries and territories. The findings from this secondary analysis reveal stark disparities in breast cancer-related disability, reflecting the complex interplay of epidemiological trends, access to care, and cultural and social factors.

The increasing burden of breast cancer worldwide, particularly in developing countries, is a call to action for enhanced global cooperation and investment in cancer control efforts. It is imperative that we address the disparities in access to high-quality services and ensure that all women with breast cancer have access to timely, effective, and culturally sensitive care.

Moving forward, it is essential to continue monitoring the burden of breast cancer-related disability and identifying opportunities for improvement. By collecting and analyzing data on a regular basis, we can track progress and identify areas where further action is needed. This includes enhancing cancer surveillance systems, improving data collection and reporting, and strengthening research on the impact of breast cancer on disability.

Our findings support the implementation of the WHO Global Breast Cancer Control Initiative, and emphasize that a “one-size-fits-all” global breast cancer control strategy is not applicable. Countries should formulate targeted prevention, screening, treatment and rehabilitation strategies based on their SDI level, age-specific burden characteristics, and health system capacity. Global health cooperation should prioritize supporting low-income countries to overcome core barriers in breast cancer care, so as to reduce the global disparities in breast cancer disability burden.

In conclusion, the findings from this secondary analysis underscore the urgent need for a comprehensive and inclusive approach to breast cancer prevention, treatment, and support. By working together to address the diverse challenges presented by breast cancer worldwide, we can improve outcomes for patients and reduce the overall burden of this disease on society.

## Supplementary Information

Supplementary Material 1: Figure S1. Age-specific rates of breast cancer-related years lived with disability (YLDs) in 2021. (A) YLDs rate among individuals aged 15–49 years. (B) YLDs rate among individuals aged 50–74 years. (C) YLDs rate among individuals aged 75 years and older.

Supplementary Material 2: Figure S2. Trends in age-standardized rates of breast cancer-related years lived with disability (YLDs) globally and across 21 GBD regions, by SDI, 1990–2021. Values are presented as age-standardized rates with corresponding 95% uncertainty intervals.

Supplementary Material 3.

Supplementary Material 4.

## Data Availability

The datasets used and/or analyzed during the current study are available from the corresponding author on reasonable request.

## Abbreviations

BC: Breast Cancer
GBD: Global Burden of Disease1
UIs: Uncertainty intervals
SDI: Socio-demographic index
AAPC: Average annual percentage changes
DALYs: Disability-adjusted life years
COVID-19: Corona Virus Disease 2019
YLDs: Years lived with disability
WHO: World Health Organization
IARC: International Agency for Research on Cancer
UN: United Nations
GATHER: Guidelines for Accurate and Transparent Health Estimates Reporting
